# A Multi-Head UNet++ Framework with Fractional Differential Output Refinement for UAV Multispectral Crop Stress Mapping

**DOI:** 10.3390/s26103228

**Published:** 2026-05-20

**Authors:** Çağrı Suiçmez, Cemal Yılmaz, Hamdi Tolga Kahraman, Yusuf Sönmez

**Affiliations:** 1 Electrical Electronic Engineering, Faculty of Technology, Gazi University, 06000 Ankara, Turkey; cemal@gazi.edu.tr; 2 Graduate School of Natural and Applied Sciences, Gazi University, 06000 Ankara, Turkey; 3 Industrial Engineering, Faculty of Engineering, Karadeniz Technical University, 61000 Trabzon, Turkey; htolgakahraman@ktu.edu.tr; 4 Computer Engineering, Faculty of Technology, Gazi University, 06000 Ankara, Turkey; ysonmez@gazi.edu.tr

**Keywords:** multispectral semantic segmentation, multi-source dataset harmonization, fractional anisotropic diffusion, physics-informed deep learning, precision agriculture, UAV-based remote sensing

## Abstract

**Highlights:**

**What are the main findings?**

- First unified five-class pixel-level modeling of abiotic water stress and biotic rust disease using UAV-based multispectral orthomosaics.
- The proposed multi-head WF-UNet++ with FPDE refinement achieves 0.8623 mIoU and 0.8439 FG-IoU, outperforming strong baselines such as UNet, DeepLabV3+, and SegFormer.

**What are the implications of the main findings?**

- Demonstrates the feasibility of harmonizing heterogeneous UAV multispectral datasets representing abiotic water stress and biotic rust disease within a common semantic segmentation framework.
- Provides a methodological basis for scalable multi-source crop stress mapping by combining multispectral data, multi-head segmentation, and fractional differential output refinement.

**Abstract:**

This study presents a unified semantic segmentation framework for UAV-based multispectral crop stress mapping, focusing on the integration of water stress and rust disease conditions within a common label space. Unlike conventional approaches that address individual stress factors independently, the proposed framework harmonizes heterogeneous datasets with different annotation schemes into a single multi-class segmentation problem. To achieve this, UAV multispectral orthomosaics are processed using a patch-based strategy and a multi-head UNet++ architecture incorporating segmentation, edge-aware, and Signed Distance Transform (SDT) branches. In addition, a physics-informed output-space refinement module based on fractional partial differential equations (FPDE) is introduced to enhance spatial coherence and boundary preservation in the predicted maps. Experimental results demonstrate the effectiveness of the proposed framework within the evaluated dataset setting, particularly in terms of boundary delineation, spatial consistency, and minority-class detection. The study highlights the feasibility of integrating heterogeneous stress conditions into a unified segmentation framework and provides a foundation for future research on scalable multi-source agricultural monitoring systems.

## 1. Introduction

In precision agriculture, remote sensing technologies are becoming crucial instruments for tracking plant health, allowing for increased output, early loss diagnosis, and more effective resource use. It is generally known that biotic and abiotic stressors, which have a direct impact on plant growth and eventually yield, account for a sizable amount of agricultural production losses. Recent years have seen significant advancements in plant stress identification thanks to the combination of deep learning techniques with remote sensing data. Nevertheless, the majority of current research examines several stressors separately, such as illness and water stress [1]. Because varied stress types from various orthomosaic sources with inconsistent semantic structures are not jointly considered within a unified framework, this leads to a substantial research gap. As a result, harmonizing heterogeneous stress datasets with different semantic structures under a unified segmentation framework remains a challenging task. Key drawbacks of deep learning-based plant stress identification techniques, such as inconsistent data, class imbalance, and generalization problems, have also been brought to light by recent review studies [2].

Convolutional neural networks in particular have proven to be highly capable of learning discriminative visual characteristics from massive datasets [3,4]. These developments have been expanded to semantic segmentation problems with the advent of fully convolutional networks (FCNs), which allow pixel-wise categorization [5]. The UNet architecture and its variants have been widely used in image segmentation tasks due to their encoder–decoder structure and skip connections, which enable the fusion of low-level spatial details with high-level semantic representations [6,7]. Furthermore, deep architectures are now more trainable and consistent thanks to residual connections and multi-scale contextual modeling techniques [8,9]. However, the majority of research in agricultural settings, such as maize fields, concentrates on one kind of stress [10,11]. Because of this, current methods frequently fall short in capturing small-scale stress regions, adapting to heterogeneous stress-related datasets, and resolving class imbalance problems in complex agricultural imagery.

High-resolution photography is even more crucial for semantic segmentation in agricultural settings due to the extensive use of UAV technology [12,13]. The modeling of spectrum responses intimately associated with plant physiology is made possible by the use of multispectral channels, offering important data for stress analysis. Nevertheless, the majority of current research relies on a single problem formulation or data source [14]. Dispersion effects are introduced by variations in sensor properties, flying conditions, and ambient factors, which hinder model generalization and cause domain shift issues [15,16,17]. As a result, combined modeling of orthomosaic data from several sources is still a challenge.

Modeling several stress sources under a single semantic segmentation framework continues to provide considerable difficulties. Deep learning-based disease identification and segmentation has made significant strides recently, especially in detecting lesion morphology and spectrum fluctuations under field circumstances [18]. Nevertheless, the majority of current methods concentrate on specific stressors or disease groups, which restricts their usefulness for unified agricultural monitoring scenarios involving heterogeneous stress-related datasets. Furthermore, current deep learning-based segmentation methods typically rely directly on network outputs without explicitly enforcing spatial consistency, which often leads to fragmented and noisy predictions in complex environments characterized by heterogeneous structures and spectrally overlapping classes.

In order to overcome these constraints, this work presents a fixed-parameter, physics-inspired output-space refinement method based on fractional-order diffusion, which allows explicit control over boundary preservation and spatial coherence during the segmentation process. The suggested paradigm allows for consistent representation across diverse datasets by modeling biotic (rust disease) and abiotic (water stress) aspects inside a single five-class semantic space. Here, conflicting label sets from several orthomosaic sources are rearranged into a single semantic framework that includes classes for rust disease, soil, healthy vegetation, mild stress, and high stress.

In this study, the proposed architecture is referred to as WF-UNet++. The abbreviation WF denotes the two main design principles of the framework: “W” refers to the wavelet-enhanced feature representation used to improve multi-scale spatial and frequency-sensitive feature extraction, whereas “F” refers to the fractional-order output-space refinement based on FPDEs. Therefore, WF-UNet++ represents a UNet++-based multi-head segmentation framework that jointly integrates wavelet-guided feature learning and fractional diffusion-based probability refinement.

The proposed approach combines class-aware loss functions, balanced patch-based training, and a multi-head UNet++ architecture. Furthermore, a curriculum learning approach is used to integrate a physics-informed output refinement module based on fractional-order anisotropic diffusion into the network. Improved modeling of multi-scale stress patterns and heterogeneous field conditions, which are challenging to capture with traditional designs, is made possible by this approach.

The main contributions of this study are summarized as follows:

- A unified semantic segmentation framework is proposed for harmonizing heterogeneous UAV multispectral datasets representing different crop stress conditions (water stress and rust disease) into a common multi-class label space.
- A multi-head UNet++ architecture is developed, integrating segmentation, edge-aware, and Signed Distance Transform (SDT) branches to improve structural consistency and boundary delineation in stress region mapping.
- A physics-informed output-space refinement module based on fractional partial differential equations (FPDE) is introduced to enhance spatial coherence and reduce noise in the predicted probability maps.
- A rule-based ground-truth generation strategy combining multiple vegetation indices (e.g., NDVI, NDRE, SAVI) and spatial post-processing is designed to enable scalable label generation from UAV multispectral data.
- The study establishes a semantic unification framework for integrating heterogeneous stress datasets rather than modeling simultaneous multi-stress interactions within a single scene.

The remainder of this article is organized as follows. Section 2 reviews related studies on crop stress detection, disease segmentation, and remote sensing–based agricultural image analysis. Section 3 presents the materials and methods used in this study, including the UAV-based multispectral datasets, the dataset harmonization process, patch-based data preparation strategy, and the proposed WF-UNet++ architecture. This section also details the multi-head design incorporating segmentation, edge-aware, and Signed Distance Transform (SDT) branches, as well as the FPDE-based output refinement module and its curriculum-based integration. Section 4 describes the training strategy, loss functions, experimental setup, and evaluation protocol. Section 5 reports the experimental results, including comparisons with state-of-the-art methods, class-wise quantitative analysis, qualitative visualizations, and ablation studies evaluating the contribution of each architectural component. Section 6 provides a discussion of the results, including computational considerations, generalization aspects, and practical implications for UAV-based agricultural monitoring. Finally, Section 7 concludes the paper by summarizing the main findings and outlining directions for future research.

## 2. Related Work

The existing image-based segmentation methods used in maize cultivation are summarized in Table 1, comparing them based on the type of phenomenon being addressed, stress nature (abiotic/biotic), description of the task, data source used, class definition, and performance metrics used. From Table 1, it can be seen that most existing works only address a particular problem (such as weed detection, leaf diseases, or plant structure-related tasks) and typically address abiotic and biotic stress sources separately. Nevertheless, based on the deep learning models used and the IoU/mIoU values achieved, it is evident that pixel-level multi-class and field-level comprehensive stress modeling has not been addressed at all in the existing literature. Based on this, the proposed work is positioned in Table 1 as a unified semantic segmentation framework that harmonizes UAV multispectral orthomosaic datasets representing graded water stress and rust disease within a common five-class label space.

Analysis of Table 1 shows that image-based methods in the current literature are mainly centered on a single phenomenon in maize production. Most of the current literature is organized around the scope of isolated problems such as weed classification, leaf diseases, and structural analysis. Moreover, abiotic and biotic factors are mainly assessed in the context of independent scenarios. This does not provide a proper representation of the complex nature of field conditions. In actual field settings, plants are generally subjected to more than one stress factor simultaneously, and these interactions have a direct impact on the spatial distribution of plant health. Nevertheless, the harmonization of heterogeneous UAV multispectral datasets representing graded water stress and rust disease into a common pixel-level semantic segmentation framework has rarely been addressed in the literature.

Methodologically, most of the studies in the literature are mainly based on conventional convolutional neural networks or U-Net and its variants. The studies are mainly focused on the diversification of architectural patterns, data augmentation, or optimization strategies. However, these studies show limited capability in terms of spatial continuity in segmentation maps, the integrity of class boundaries, and particularly the modeling of physiological transitions between different levels of stress. In most of the studies in the literature, the output maps generated by the network are directly accepted as the final output. The modification steps that would give meaning to the output maps in a biophysical sense or enhance mathematical consistency are often neglected. This can result in noisy, fragmented, or spatially inconsistent segmentation maps, particularly in field scenes with heterogeneous structures and complex patterns.

One of the most obvious inconsistencies is observed in the current literature available for experimental evaluation. Although some literature focuses on the performance of segmentation tasks using conventional metrics like IoU or mIoU at the pixel level, most of the literature available focuses only on accuracy, F1-measure, or regression scores. This creates a problem in objectively assessing the performance of approaches for semantic segmentation tasks. However, most of the literature available is restricted to binary classification problems. The impact of multi-class and imbalanced data distributions on the performance of approaches is not properly addressed. The absence of class-level performance analysis, particularly in complex field conditions involving multiple stresses, raises doubts about the generalizability of approaches to practical applications.

This study addresses the above-mentioned gaps by formulating a unified semantic segmentation framework for heterogeneous UAV multispectral datasets representing graded water stress and rust disease. Rather than claiming simultaneous multi-stress detection within a single field scene, the proposed approach focuses on harmonizing semantically different stress datasets into a common five-class label space. The framework extends conventional segmentation outputs by incorporating boundary- and distance-aware auxiliary supervision through a multi-head UNet++ architecture. In addition, fractional differential output refinement is used to improve spatial coherence and preserve transition structures in the predicted probability maps. The experimental evaluation is conducted using class-wise IoU and FG-IoU metrics under a multi-class and imbalanced dataset setting. In this context, the study contributes a controlled multi-source semantic unification strategy for UAV-based crop stress mapping.

## 3. Materials and Methods

**Figure 1:**
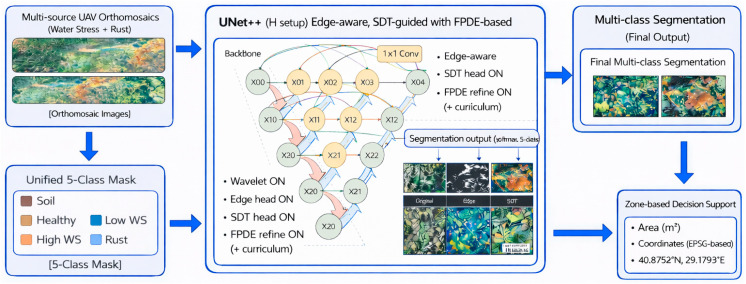
Graphical summary of proposed methods.

The methodological workflow of this study was designed to transform two semantically different UAV multispectral orthomosaic datasets into a unified five-class semantic segmentation problem. First, water stress and rust disease orthomosaics were harmonized into a common label space consisting of soil, healthy vegetation, low water stress, high water stress, and rust disease. Second, pixel-level ground-truth masks were generated using vegetation-index-driven decision rules and converted into overlapping 224 × 224 image–mask patches for model training. Finally, the proposed WF-UNet++ framework was trained with multi-head supervision and FPDE-based output-space refinement to improve boundary localization, spatial coherence, and minority-class segmentation performance. The overall methodological pipeline is summarized in Figure 1.

Figure 1 provides a holistic overview of the end-to-end operation of the proposed multi-stage analysis pipeline. This structure systematically presents the process, starting from multi-source UAV orthomosaics and extending to the generation of field-scale decision support outputs. In the first stage, different orthomosaic datasets for water stress and rust disease are combined in a common semantic space. This process makes a single mask with five categories: soil, healthy plants, low water stress, high water stress, and rust disease. This reduces semantic inconsistencies and enables harmonized learning from heterogeneous stress-related datasets within a unified segmentation paradigm. This approach reduces discrepancies that arise between various models, providing a more cohesive view. The WF-UNet++ model analyzes the unified data structure in the second stage. This model is enhanced using wavelet multi-scale features, edge-aware convolution, Signed Distance Transform guidance, and an FPDE-based enhancement module. The aim is to provide greater accuracy, particularly in edge regions, and to more effectively distinguish small or irregular stress regions. Wavelet representation enhances detail detection capability over a broad range of frequency scales. SDT guidance enhances our boundary detection learning. The FPDE module reduces noise and enhances spatial continuity, making it simpler to generalize. Finally, the results of the multi-class segmentation are projected back into the orthomosaic plane. This enables the derivation of decision support indicators such as class distribution ratios, affected area size, and geographic location. Consequently, field-scale interpretation of patch-based deep learning outputs is possible. For precision agriculture applications, the proposed method thus yields pixel-level segmentation along with an integrated analysis framework that converts these outputs into actionable, comprehensible, and quantifiable decision support metrics.

### 3.1. Dataset Description

High-resolution multispectral orthomosaic images acquired via UAV-based remote sensing comprise the datasets used in this investigation [39].

To provide a comprehensive understanding of the datasets and their spatial characteristics, the geographical context of the study area and representative UAV multispectral orthomosaic views are jointly presented. Since water stress and rust disease originate from different physiological mechanisms, their spatial distribution patterns and visual manifestations exhibit distinct characteristics at both the field scale and local canopy level. Therefore, a combined visualization strategy is adopted to illustrate not only the overall orthomosaic structure but also localized regions where stress patterns are more clearly observable.

**Figure 2:**
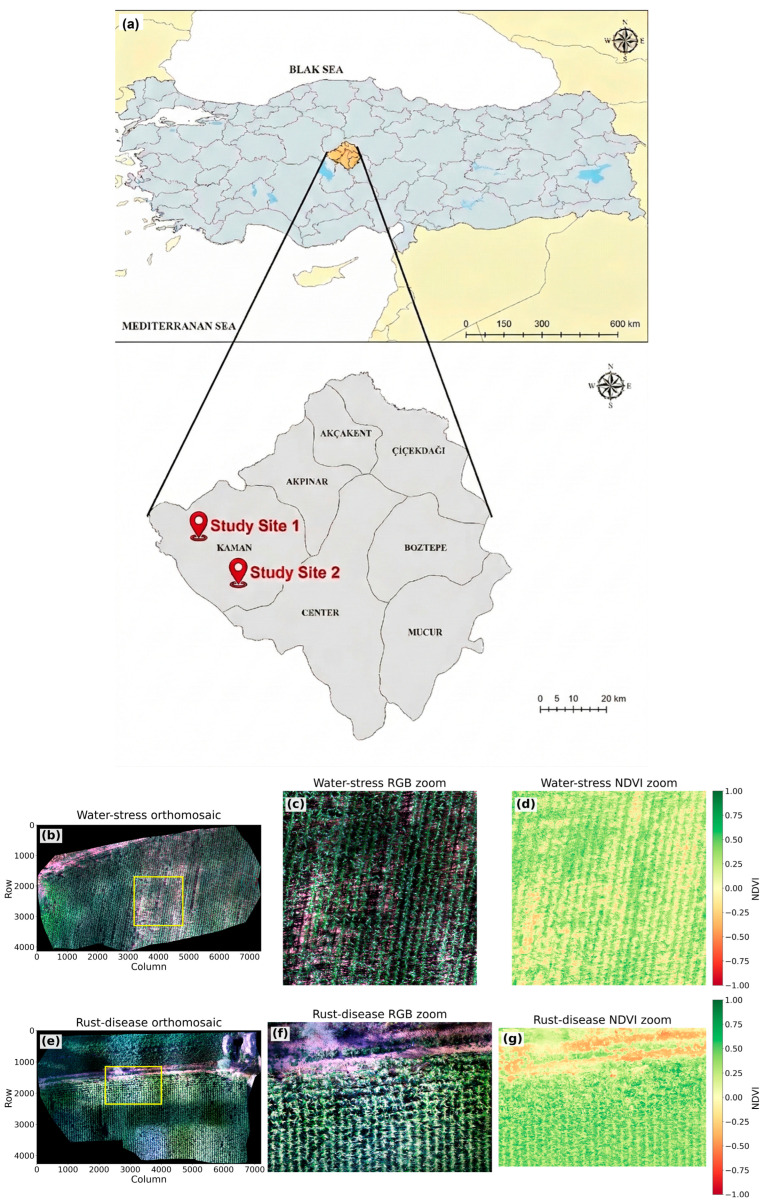
Geographical location of the study area and representative UAV multispectral orthomosaic views with localized enlarged RGB and NDVI panels. (**a**) Location of the two maize production fields in Kırşehir, Turkey. (**b**) Water stress orthomosaic with the selected zoom region. (**c**) Localized RGB zoom of the water stress region. (**d**) NDVI zoom of the same water stress region. (**e**) Rust disease orthomosaic with the selected zoom region. (**f**) Localized RGB zoom of the rust disease region. (**g**) NDVI zoom of the same rust disease region. The yellow boxes in panels (**b**,**e**) indicate the selected regions that were enlarged and visualized as the corresponding RGB and NDVI zoom panels. The enlarged panels highlight the gradual and spatially continuous canopy degradation associated with water stress and the more localized and clustered degradation pattern associated with rust disease.

As shown in Figure 2b–d, water stress manifests as a more gradual, heterogeneous, and spatially continuous canopy degradation pattern across the field. In contrast, as illustrated in Figure 2e–g, rust disease exhibits more localized and clustered regions characterized by sharper visual and structural degradation. To further clarify these differences, localized enlarged RGB views (Figure 2c,f) are provided, highlighting canopy-level structural variations between the two stress types. In addition, NDVI-based zoom panels (Figure 2d,g) are included to support this distinction from a spectral perspective. These panels demonstrate that water stress leads to more diffuse and continuous reductions in vegetation vigor, whereas rust disease produces more spatially concentrated and abrupt decreases in NDVI values. This combined visual and spectral representation enables a clearer interpretation of the distinct spatial signatures associated with abiotic and biotic stress conditions.

### 3.2. Dataset Harmonization and Class Definition

The dataset used in this study includes labels derived from two different orthomosaics and created according to different problem definitions. The first orthomosaic is based on a multi-class structure representing different severity levels of water stress in vegetation. The second orthomosaic focuses on identifying infection sites of rust disease in maize plants. Both data sources provide important information for agricultural decision support. However, their label structures are not directly compatible in terms of class numbers and semantic content. Therefore, it is not possible to use the raw datasets together under a single, integrated deep learning model.

Most orthomosaic-based semantic segmentation studies in the current literature focus on a single type of stress. These studies are generally conducted with limited data sources and often use binary classification approaches. However, practical agricultural monitoring systems should be able to interpret different abiotic and biotic stress conditions within a consistent semantic framework, even when such conditions are represented by heterogeneous data sources. Accurate differentiation of these situations is critical for decision support systems in precision agriculture. In this study, to address this gap in the literature, tag sets obtained from different orthomosaics have been redefined. The tags have been harmonized consistently under a common semantic space.

In the labeling process, ground areas common to both datasets were preserved as the Soil class. Pixels of vegetation were reclassified based on the degree and status of stress. Healthy vegetation was defined as areas showing healthy growth. Two groups of water-stressed areas were created: Areas with low densities were designated as low stress, while areas with high densities were designated high stress. Since rust disease is a biotic process, affected areas were classified as a distinct class of rust disease. A consistent five-class semantic space that works with all datasets was produced by this restructuring. At the pixel level, this structure is able to differentiate not only between areas that are stressed and those that are healthy, but also the kind and intensity of stress. As a result, the model can learn not only whether stress is present but also how much of it there is and whether it is linked to a disease. This provides more functional outputs for targeted intervention plans in precision agriculture. To effectively utilize the five-class structure, images were processed with a patch-based representation. Although full orthomosaics cover large areas, stress indicators mostly consist of small and scattered structures. Therefore, tile-based and patch-based learning approaches were adopted. The goal is to enable the model to better learn both class diversity and small-scale stressors.

In this study, orthomosaic images were processed by dividing them into patches of 224 × 224 pixels. This patch size allows for the representation of small stress and disease regions without loss of detail, while preserving sufficient spatial context. An overlapping splitting strategy was used in the patch extraction process. This approach prevents pixels at class boundaries from being limited to a single patch. It allows the same pixels to be represented again in the model in different contexts. Thus, it supports the learning of spatially limited and unevenly distributed classes such as low stress, high stress, and rust disease. As a result, the overall class discrimination of the model is increased.

A significant advantage of patch-based representation is that it partially reduces the class area imbalance frequently seen in agricultural imagery. In field scenes, Healthy vegetation and Soil cover large areas, while low stress, high stress, and rust disease classes are often concentrated in small regions. This imbalance makes it difficult to learn these small classes during training across the entire image. Thanks to the patch-based structure, small but critical regions are more frequently included in training. Thus, the model’s capacity and sensitivity to learning these classes are significantly increased.

In conclusion, the label matching and redefined class structure developed in this study make it possible to use different orthomosaic sources together under a single deep learning model. The overlapping patch-based data representation supports more effective learning of small, scattered, and rare stress regions by the model. The data structure and semantic class space defined in this section form a foundation for the model architecture and training strategies that will be explained in subsequent sections.

### 3.3. Ground Truth Generation and Dataset Preparation

The ground-truth masks used in this study were generated at the pixel level from six-band UAV orthomosaics. These masks were then converted into a patch-based structure compatible with model training. NoData pixels and potential digital overflows were considered during masking and data preparation processes. All operations were performed using a valid mask that identifies only valid fields. Therefore, only pixels with finite values and no NoData were included in the labeling process. This ensured that the model was exposed only to meaningful and reliable data.

**Figure 3:**
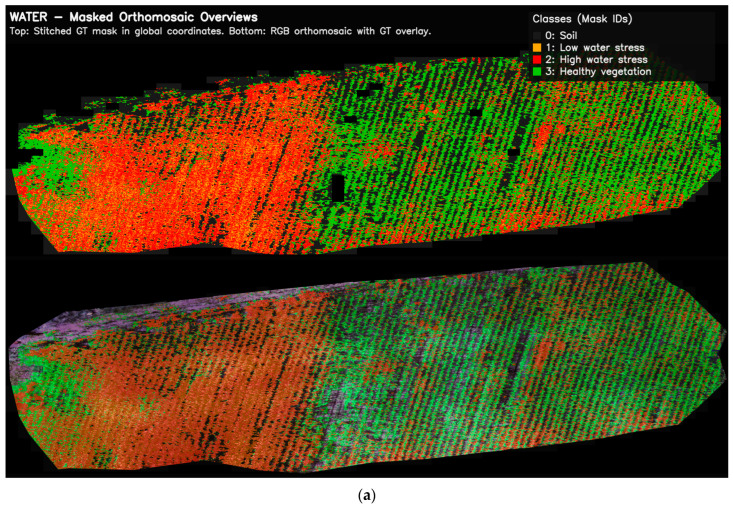

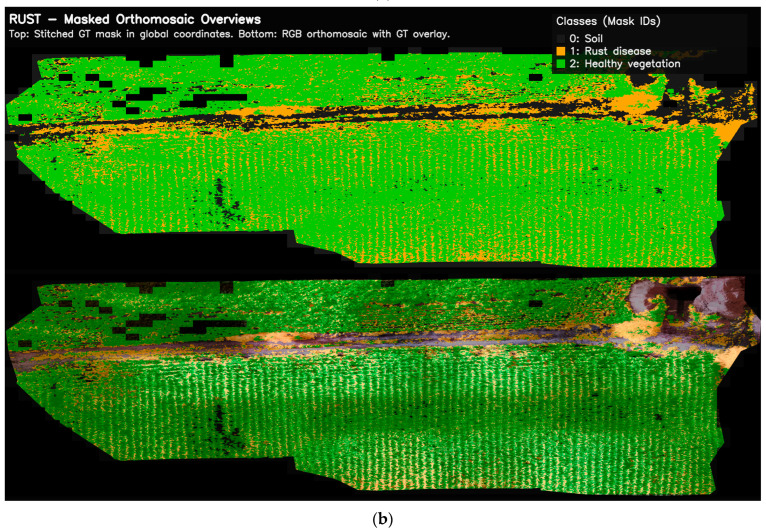
Orthomosaic-level visualization of the generated ground-truth masks for the two UAV datasets. (**a**) Water stress dataset, (**b**) Rust disease dataset.

It is important that the pixel-level georeferenced masks remain consistent not only at the patch scale but also within the full orthomosaic extent. This is critical for qualitatively inspecting the labeling process. Therefore, the GT masks generated for both UAV datasets were reassembled at their original orthomosaic coordinates and visualized at a global scale. This approach allows the spatial distribution of classes to be examined and potential labeling artifacts to be identified. It also enables comparison of field-scale patterns associated with abiotic and biotic stress conditions. Thus, both patch-level plausibility and field-scale consistency of the semantic labels used for model training can be qualitatively assessed. The orthomosaic-level outputs of this verification process are presented in Figure 3.

Figure 3 shows the orthomosaic scale view of GT masks generated for two different UAV datasets. Figure 3a,b show the class labels reassembled in their original orthomosaic coordinates for the water stress and rust disease datasets, respectively. Figure 3 provides a qualitative visual inspection by overlaying the generated masks onto the corresponding RGB orthomosaics. In the rust disease dataset, infected areas are clustered along plant rows. In the water stress dataset, stress intensity shows a heterogeneous distribution within the field. These findings demonstrate that the label generation approach can capture meaningful spatial patterns not only at the pixel level but also at the field scale. Furthermore, this provides a visually plausible basis for patch-based model training in subsequent stages, although it does not replace expert-annotated ground-truth validation.

The ground-truth generation process is designed to leverage multiple spectral indicators rather than relying on a single index, thereby improving robustness against threshold sensitivity and environmental variability.

#### 3.3.1. Ground Truth Mask Generation (Water & Rust)

The water stress mask was produced by a stepwise decision chain through plant indices to separate vegetation and stress severity. First, SAVI was used for plant–soil separation and NDRE for plant vigor/stress sensitivity. The mathematical equations of the indices used are given in Equations (1) and (2).

(1) $$SAVI=\frac{\left(NIR-Red)(1+L\right)}{NIR+Red+L+\epsilon },L=0.5$$

(2) $$NDRE=\frac{NIR-RedEdge}{NIR+RedEdge+\epsilon }$$

where NIR, Red, and RedEdge denote the near-infrared, red, and red-edge band reflectance values, respectively. In the SAVI formulation, L represents the soil brightness correction factor and was set to 0.5 in this study to reduce background soil effects, while ε denotes a small positive constant used to avoid numerical instability.

Here, NIR (Near-Infrared reflectance), Red (Red band reflectance) and RedEdge (Red edge band reflectance) are represented. Vegetation is separated into “healthy-candidate” and “stressed-candidate” pixels using a second Otsu threshold via NDRE in pixels other than soil. The low vs. high distinction of stressed candidate pixels is made with two features. NDVI and GCI, mathematical index equations are given in Equations (3) and (4).

(3) $$NDVI=\frac{NIR-Red}{NIR+Red+\epsilon }$$

(4) $$GCI=\frac{NIR}{Green+\epsilon }-1$$

Here, Green represents the green band reflectance, and ε denotes a small positive constant. A feature space defined by [NDVI, GCI] was constructed for the candidate stressed pixels and partitioned into two subgroups using k-means clustering with k = 2. The resulting clusters were assigned as low and high stress, respectively, based on their NDVI characteristics. Specifically, the cluster with higher mean NDVI values was interpreted as representing lower stress, while the cluster with lower mean NDVI values was associated with higher stress. Finally, the class IDs were defined as 0: soil, 1: low stress, 2: high stress, and 3: healthy. Optionally, DenseCRF post-processing is applied to spatially refine the mask and improve boundary consistency.

The rust disease mask was generated by first distinguishing between soil and plant, and then distinguishing rust within the plant pixels. SAVI was used for soil distinction. This time, the threshold was determined using 1-dimensional 2-cluster k-means on SAVI values instead of Otsu, and the threshold was chosen to represent the smaller “soil-like” cluster from the centers.

A “voting” scheme was implemented for rust detection on plant pixels. In this stage, Simple Ratio (SR) was used in addition to NDRE and NDVI. The mathematical equation is given in Equation (5).

(5) $$SR=\frac{NIR}{Red+\epsilon }$$

In the Plant region, the median threshold was used for each index. A pixel received one vote when each of the following conditions was met: $NDRE<{\tilde{t}}_{NDRE}$, $NDVI<{\tilde{t}}_{NDVI}$, $SR<{\tilde{t}}_{SR}$. Pixels with a total vote ≥ 2 were assigned as rust, and the mask was cleaned using morphological filtering operations. The end-class IDs are 0: soil, 1: rust, 2: healthy. DenseCRF can also be applied optionally to this mask.

This script generates masks for the water and rust datasets in their respective task spaces. The water dataset has four classes, and the rust dataset has three. These masks are merged into a common area in the mix scenario, which is where the last model training is carried out. The water masks maintain the soil, low stress, high stress, and healthy classes. According to the rust masks, the rust class is a fifth class with a distinct identity. Additionally, the common healthy vegetation class is mapped to the healthy class in the rust dataset. Soil, healthy vegetation, low stress, high stress, and rust disease classes make up the five-class semantic space that is thus produced for combined training.

Representative NDVI and NDRE maps used during the ground-truth generation process are provided in Appendix A. These supplementary maps are included to support the visual verification of the spectral-index-based labeling procedure. They are placed in the Appendix A rather than the main text to preserve the readability of the methodological flow while still providing additional evidence for the vegetation vigor and stress-related spectral patterns used during mask construction.

**Figure 4:**
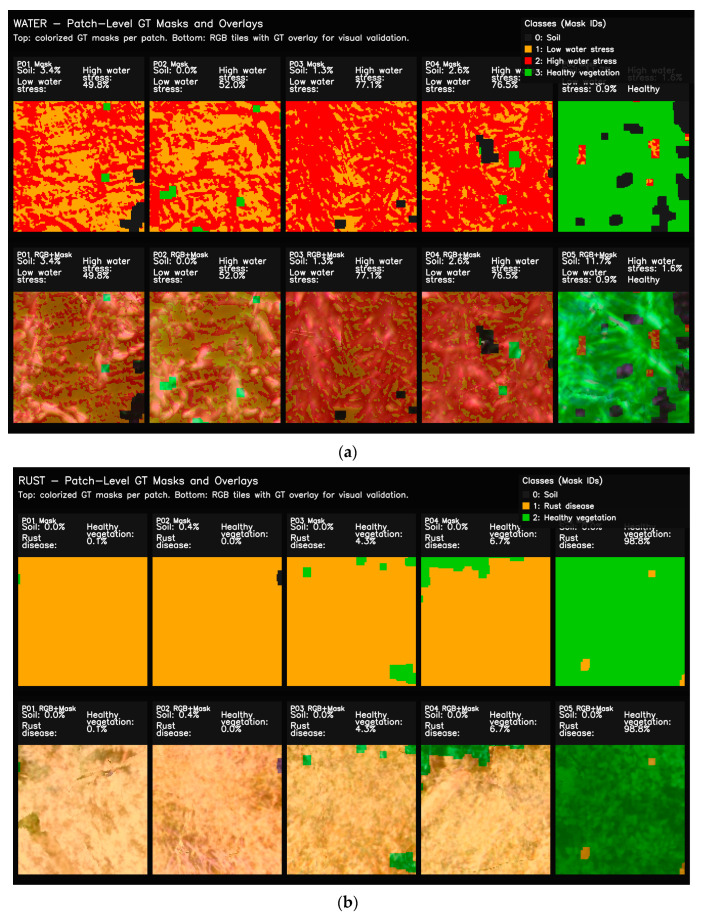
Patch-level ground-truth (GT) masks and RGB overlays. (**a**) Colored GT masks (top row) and corresponding RGB-mask overlaps (bottom row) showing class distributions in sample patches of the water stress dataset. (**b**) Colored GT masks (top row) and corresponding RGB-mask overlaps (bottom row) in sample patches of the rust disease dataset.

To investigate how patch-based data representation influences model learning, ground-truth masks from both datasets were visualized at the patch scale. This approach enables the assessment of class distributions not only across the entire orthomosaic but also within the individual samples used during training. In particular, ensuring the proper representation of small, fragmented, and heterogeneous classes such as water stress and rust disease within the patches is essential for accurately evaluating the model’s pixel-level discrimination capability. To support this analysis, colored ground-truth masks and RGB-mask overlays are presented together for selected representative patches, allowing visual verification of label correctness and spatial consistency. Representative examples of this patch-level class structure and validation are illustrated in Figure 4.

Figure 4 illustrates that, in the water stress dataset, the low- and high-stress classes are distributed across patches as interwoven, irregular, and fine-scale structures, whereas healthy vegetation forms more homogeneous regions. In the rust disease dataset, infected areas emerge as spatially limited yet high-density clusters that are clearly separated from surrounding healthy tissue. RGB-mask overlays provide a qualitative inspection of the automatically generated GT masks and suggest visually plausible spatial agreement with the corresponding canopy patterns. However, these visual overlays should not be interpreted as a substitute for expert-annotated ground-truth validation. Furthermore, these patch samples show that class imbalance is partially reduced at the patch scale compared to the image scale. It appears that rare classes become more visible during the training process. Thus, it is clearly seen that patch-based representation offers a fundamental mechanism that strengthens the learning of small-scale stress and disease regions.

#### 3.3.2. Patch Extraction Strategy

**Figure 5:**
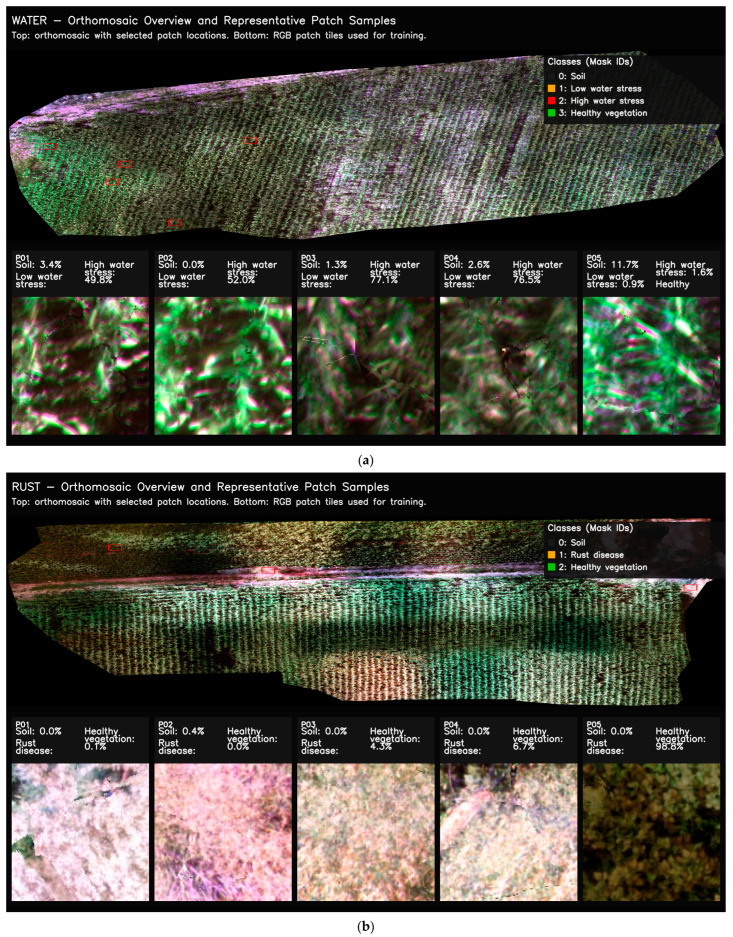
Orthomosaic-level visualization of the patch extraction strategy for the two UAV datasets. (**a**) Water stress dataset, (**b**) Rust disease dataset.

Training examples from orthomosaics were generated using a tile-based approach. For each task, the image and corresponding mask data were first normalized to a range of 0–1 within the 2–98% percentile range. Then, the orthomosaic was systematically scanned using tile × tile sliding windows. In this study, tile = 224 and stride = 96 were selected as defaults. A stride value smaller than tile naturally ensures overlapping patch generation. Directly feeding orthomosaic images into training increases memory and computational load. It also makes learning small, fragmented, and irregular stress structures more difficult. Overlapping patch-based scanning, on the other hand, allows pixels at class boundaries to be seen again in different contexts. It ensures that rare classes are represented more frequently within patches. Thus, the model’s discriminative learning capacity is strengthened. The equivalent of the applied patch extraction process at the orthomosaic level and representative patch examples used in training are presented in Figure 5.

Figure 5 shows the selected patch locations on the water stress and rust disease orthomosaics. These panels illustrate the spatial distribution of the samples included in model training across the orthomosaic extent. Figure 5 also contains representative RGB patch samples obtained from these locations. These samples provide a qualitative illustration of soil, healthy vegetation, water stress, and rust disease patterns at the patch level. After patch generation was completed, the input image and ground-truth mask for each patch were exported jointly in the same coordinate window. This created a consistent training infrastructure for subsequent operations such as dataset splitting, metadata generation, and imbalance checking.

#### 3.3.3. Dataset Splitting and Preparation (Meta, Negative Sample Control, Class Ratios)

The input image for each patch was saved as a .npy file in float16 format. The mask data was exported as a .png file containing the uint8 class ID for training purposes. Color mask outputs and RGB-mask overlaps at specific intervals were also generated for visual quality control. Negative patch control was applied to reduce data imbalance. The foreground ratio was calculated using the low and high classes in the water dataset and the rust class in the rust dataset. Patches are discarded when the foreground ratio falls below a predefined minimum threshold. To prevent negative patches from suppressing the dataset, the retention rate was limited using the keep_negative_ratio parameter.

### 3.4. Proposed Network Architecture

This study proposes a multi-headed and morphologically aware deep learning architecture based on UNet++ [40], referred to as WF-UNet++, to address the five-class semantic segmentation problem derived from multi-source multispectral orthomosaics. In this naming, “W” denotes the wavelet-enhanced representation mechanism incorporated into the network to support multi-scale and frequency-sensitive feature extraction, while “F” denotes the fractional-order FPDE-based output-space refinement module designed to improve spatial coherence and boundary-preserving smoothing in the predicted probability maps. Thus, the term WF-UNet++ explicitly reflects the two main methodological extensions added to the UNet++ backbone: wavelet-guided feature modeling and fractional diffusion-based refinement. The proposed network architecture processes patch inputs of 224 × 224 pixel size and predicts five classes—soil, healthy vegetation, low stress, high stress, and rust disease—at the pixel level. The architecture is built on an encoder–decoder backbone with dense hopping connections. It is also supported by edge-aware convolution layers and auxiliary headers based on Signed Distance Transform. To improve spatial consistency in model outputs, an output manipulation module based on fractional-order Perona–Malik anisotropic diffusion has been developed and integrated into the network. This module operates with fixed FPDE hyperparameters and multi-scale output-space refinement settings. It is gradually integrated into the training process using a curriculum learning approach. Thus, the learning process becomes more stable and directed.

**Figure 6:**
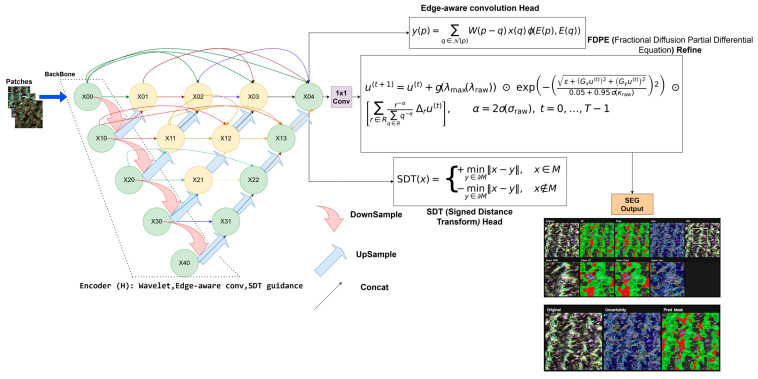
Architecture of the proposed model.

Figure 6 visually presents the general architecture of the proposed model. The proposed model is built upon a UNet++-based backbone designed to process multispectral patch inputs. In the encoder stage, wavelet-assisted, edge-aware, and SDT-guided convolutional operations are employed to capture multi-scale and structurally consistent feature representations. These features are then integrated within the decoder through the dense skip connections characteristic of UNet++, enabling the reconstruction of high-resolution outputs.

Apart from the main segmentation head, two auxiliary segmentation heads, Edge and Signed Distance Transform (SDT), are introduced to improve structural understanding during training, especially around the boundaries of classes. The probability maps of classes produced from the main segmentation head are then fed into an FPDE-based output-space refinement module, which is based on fractional partial differential equations, for spatial refinement. The final segmentation output is obtained from the refined probability maps.

The proposed model produces a single output for the final segmentation result. The edge-aware convolution head and the Signed Distance Transform (SDT) head are designed as auxiliary prediction branches in the proposed model. These branches are only active during training and add more loss terms to the model for improving edge awareness and structural sensitivity. During testing, the auxiliary outputs are not used, included in the final decision-making process, or compared with the model outputs. Their main purpose is to facilitate the learning process and improve the optimization of the primary segmentation output.

On the other hand, the Fractional Partial Differential Equation (FPDE) module does not act as a standalone prediction head. It rather works as a refinement mechanism in the output space and directly refines the class probability maps obtained from the main segmentation head. This spatial refinement helps improve the quality of the probability maps, and the final segmentation is obtained from the maps.

#### 3.4.1. Backbone Architecture

The backbone of the proposed network is a UNet++-based encoder–decoder structure [41] that aims to enhance fine boundary details while preserving multi-scale contextual information. The input is defined as a patch of size H×W×C. In this study, H=W=224 and C depends on the image representation used. The main objective of the network is to generate K=5 class possibilities for each pixel. Therefore, the final output of the network is as in Equation (6).

(6) $$\hat{Y}\in [0,\;1{]}^{H\times W\times K},\sum_{k=1}^{K}{\hat{Y}}_{i,j,k}=1$$

It is defined as shown in the equation, and class probabilities are obtained with softmax in the last layer. The mathematical equation is given in Equation (7).

(7) Y^i,j,k=exp(zi,j,k)∑t=1Kexp(zi,j,t),k∈{1,…,K}i

The encoder repeats two basic operations at each level: (i) local feature extraction (convolution blocks), (ii) downsampling the wider context by reducing the resolution. l∈{0, 1, …, L} represents the resolution level, l=0 represents the highest resolution. Feature maps in the encoder are expressed as in Equation (8).

(8) $$E^{\left(l\right)}=f_{\mathrm{enc}}^{\left(l\right)}\left(D\;\left(E^{\left(l-1\right)}\right)\right),l\geq 1,E^{\left(0\right)}=f_{\mathrm{enc}}^{\left(0\right)}(X)$$

Here, X is the input patch. D(⋅) is the downsampling operator, such as 2 × 2 max-pooling or strided convolution. $f_{\mathrm{enc}}^{\left(l\right)}(\cdot )$ typically represents successive Conv–Norm–Activation blocks. Thus, as we descend to lower levels, spatial resolution decreases, the number of channels increases, and a larger spatial context is captured.

The decoder renders the multi-scale representations learned by the encoder back to a high-resolution state. The fundamental difference in UNet++ is the use of dense and nested skip connections instead of the single skip connections of classic U-Net. This structure allows for more frequent and richer fusion of features at different depths between the encoder and decoder.

In general, an upsampling operation can be described by the following Equation (9).

(9) $$U^{\left(l\right)}=Up\;(F^{\left(l+1\right)})$$

Here, Up(⋅) can be bilinear upsampling + convolution or transposed convolution.

In the decoder, the concatenation process is performed by concatenation along the channel axis and is shown with Equation (10).

(10) $$C^{\left(l\right)}=\mathrm{Concat}\;(U^{\left(l\right)},S^{\left(l\right)}),{\;F}^{\left(l\right)}=f_{\mathrm{dec}}^{\left(l\right)}\;(C^{\left(l\right)}),$$

Here, $S^{\left(l\right)}$ represents the skip link(s) at the relevant resolution.

The distinguishing feature of UNet++ is that instead of a single skip from the encoder at each resolution level, it creates enriched skip paths through intermediate nodes. In the literature, the common notation $X^{i,j}$ represents the resolution level i and j represents the “nesting” depth at the same level. $X^{i,0}$ is the direct feature from the encoder (base node), $X^{i,j}$ (j≥1) is the node obtained by combining the previous intermediate nodes and features carried up from the lower level. The UNet++ union rule can be written as in Equation (11).

(11) $$X^{i,j}=f^{i,j}(\mathrm{Concat}(X^{i,0},X^{i,1},\ldots ,X^{i,j-1},Up(X^{i+1,j-1}))),\;j\geq 1.$$

This equation expresses that a new node is generated by densely combining previous intermediate representations ($X^{i,0}\ldots X^{i,j-1}$) at the same resolution and the representation sampled up from a lower resolution ($Up(X^{i+1,j-1})$). Thus, the mesh progressively blends both the fine detail information of shallow layers and the contextual information of deep layers; strengthening pixel separation in agricultural scenes where boundaries are blurred.

Class logits are generated by 1 × 1 convolution over the final representation obtained at the highest resolution $X^{0,J}$. The mathematical equation is given in Equation (12).

(12) $$z={\mathrm{Conv}}_{1\times 1}(X^{0,J}),z\in R^{H\times W\times K},$$

Softmax calculates 5 class possibilities at the pixel level. This backbone provides a robust multi-scale basis representation for the auxiliary headers (edge-aware and SDT) and output manipulation (FPDE) module, which are defined in the following subheading.

#### 3.4.2. Multi-Head Design

The proposed architecture extends the multi-scale feature representations derived from the UNet++ backbone with a multi-head structure capable of learning boundary and geometry information as well as pixel-level segmentation. Based on the highest-resolution combined representation of the backbone, $F\in R^{H\times W\times C}$ ($X^{0,J}$ in Section 3.4.1), three parallel outputs are generated: (i) main segmentation header, (ii) edge-aware header, and (iii) Signed Distance Transform (SDT) header. This multi-tasking design aims to better organize the decision surface and improve spatial consistency in agricultural scenes where inter-class boundaries are unclear (especially in stress/rust zones).

(i) Main Segmentation Module (Segmentation Head)

The segmentation head is responsible for pixel-level classification by converting the multi-scale feature representations learned by the backbone into class probabilities for each pixel. Deep learning-based semantic segmentation has become the dominant approach for dense prediction problems today due to its capacity to learn hierarchical representations and provide high accuracy on large and complex datasets. This is detailed in comprehensive reviews of current studies in the literature [42].

The main module generates the probability distribution of K=5 classes for each pixel. The logit map $z\in R^{H\times W\times K}$ is obtained with 1 × 1 convolution. Equations (6) and (12) are given. Thus, the segmentation output Y^∈[0, 1]H×W×K is obtained.

(ii) Edge-Aware Head

To sharpen the segmentation boundaries and increase the discrimination power in the class transition zones, the second heading generates an edge probability map at the pixel level. The edge heading makes a binary prediction over the same backbone representation [43]. The mathematical equation is given in Equation (13) and (14).

(13) $$e={Conv}_{1\times 1}(F),e\in R^{H\times W}$$

(14) E^=σ(e)

Here, σ(⋅) is the sigmoid function and E^∈(0,1)H×W represents the edge probabilities. The ground truth map E for the edge is usually derived from the segmentation mask Y. In this study, a morphological gradient definition given in Equation (15) can be used to capture the class boundaries.

(15) $$E=\delta (Y)-\varepsilon \left(Y\right)$$

Here, δ(⋅) and ε(⋅) are the dilation and erosion operators, respectively. In the multi-class case, Y is a “label image” and this operation marks the class transitions as boundary bands. Alternatively, edges can be extracted and combined from the binary mask $Y^{\left(k\right)}$ for each class. The following Equation (16) explains this.

(16) $$E=\sum_{k=1}^{K}\left(\delta (Y^{\left(k\right)})-\epsilon (Y^{\left(k\right)})\right)$$

This head, together with the training losses described in the following section, encourages the learning of more distinctive features, particularly along class boundary regions.

(iii) Signed Distance Transform Module (SDT Head)

The Signed Distance Transform (SDT) module produces a regression output inspired by distance-based boundary representations, aiming to model class boundaries with a continuous spatial representation. This approach aims to improve boundary accuracy in segmentation outputs by enabling more stable learning of sharp class transitions at the pixel level [44].

First, let a boundary set ∂Ω be defined from the segmentation locality accuracy. Let d(p,∂Ω) be the Euclidean distance of a pixel from the boundary, and the signed distance function is as in Equation (17).

(17) $$D(p)=\left\{\begin{array}{c} +d(p,\partial \Omega ),\\ -d(p,\partial \Omega ), \end{array}\right.\begin{array}{cc} & p\in \Omega \\ & p\notin \Omega \end{array}$$

Here, Ω represents the target region (e.g., plant or specific class region). In the case of multiple classes, the SDT can be handled in two ways: (i) taking all class boundaries as a single combined boundary and generating a single-channel SDT, (ii) generating separate SDTs for each class. In this study, a single-channel SDT representation is preferred in terms of computational efficiency and generalization. The output of the SDT header is given in Equation (18).

(18) D^=g(F),D^∈RH×W

Here, g(⋅) is the regression header containing several convolution layers and a 1 × 1 projection on the backbone representation. In practice, SDT values can be scaled and trimmed to a specific range as in Equation (19) to prevent large distances from becoming dominant.

(19) $$\overset{\sim }{D}(p)=clip(\frac{D(p)}{\tau },-1,1)$$

Here, τ is the scale parameter. The model learns this normalized target via $\overset{\sim }{D}$. As a result, the model produces the output set in Equation (20) via the backbone representation F.

(20) O={Y^,E^,D^}

This design aims to generate more stable and spatially consistent predictions on subtle class transitions and small stress zones observed in agricultural orthomosaics by learning (i) the class separation of the main segmentation header, (ii) the boundary sharpness of the edge header, and (iii) the boundary geometry of the SDT header together. The next section presents an FPDE-based output enhancement module that further refines these outputs spatially.

#### 3.4.3. Fractional-Order Anisotropic Diffusion Refinement (FPDE)

Pixel-level class probabilities generated by deep networks can contain spatial inconsistencies, particularly due to noisy textures, weak class boundaries, and small-scale stress zones observed in agricultural scenes. This study proposes an output refinement module (FPDE) based on a fractional-order generalization of the classical Perona–Malik anisotropic diffusion model [45] to spatially refine network outputs and strengthen boundary continuity. The FPDE module is a refinement mechanism that operates not on the network’s feature maps, but on the class probability maps generated by the main segmentation header, i.e., in the output space.

(i) Classical Anisotropic Diffusion

The Perona–Malik model [46] enables edge-sensitive diffusion propagation over a scalar field u(x,t) and is described by the partial differential Equation (21).

(21) $$\frac{\partial u}{\partial t}=\nabla \cdot (c(|\nabla u|)\nabla u),$$

Here, ∇ represents the gradient operator, ∇⋅(⋅) the divergence, and c(⋅) the diffusion coefficient. The function c(∣∇u∣) ensures boundary preservation by suppressing diffusion in regions with high gradients (edges). The general equations are given in Equation (22).

(22) $$c(|\nabla u|)=\exp\left(-\frac{|\nabla u{|}^{2}}{\kappa^{2}}\right)\mathrm{or}\;c(|\nabla u|)=\frac{1}{1+{\left(\frac{|\nabla u|}{\kappa }\right)}^{2}}$$

Here, κ is a parameter that controls the edge sensitivity.

(ii) Fractional Order Generalization

Classical second-order diffusion relies on local neighborhoods and captures long-range relationships to a limited extent [47]. In this study, a fractional-order generalization of the Laplacian operator is used to obtain a more flexible spatial arrangement. The fractional diffusion equation is expressed in Equation (23).

(23) $$\frac{\partial u}{\partial t}=-\lambda \;(-\Delta {)}^{\alpha /2}u,\;0<\alpha \leq 2$$

Here, $(-\Delta {)}^{\alpha /2}$ represents the fractional Laplacian operator and α is the parameter determining the order of diffusion. Classical Laplacian diffusion is obtained when α=2 while a longer-range and smoother diffusion behavior emerges when α<2. This feature allows small and fragmented stress regions to become more contextually consistent.

(iii) FPDERefine Module (Application on Network Outputs)

The proposed FPDERefine module combines the concepts of anisotropic and fractional diffusion and is applied to the output maps generated by the network. For the class probabilities $P_{k}(x)$ obtained from the main segmentation header, each class channel k is treated independently and as shown in Equation (24) and (25).

(24) $$u_{k}^{\left(0\right)}\left(x\right)=P_{k}\left(x\right),k=1,\ldots ,K$$

In each iteration, the output is updated as follows. Here, $P_{k}(x)$ represents the pixel-level probability map of class k obtained from the softmax output of the main segmentation header, and $u_{k}^{\left(t\right)}(x)$ represents the refined probability map in the t-th iteration of the FPDERefine module. x, indicates the pixel position, and K indicates the number of classes.

(25) $$u_{k}^{\left(t+1\right)}(x)=u_{k}^{\left(t\right)}(x)+\lambda \sum_{m=1}^{M}w_{m}(\alpha )\;\nabla \cdot (c(∥\nabla u_{k}^{\left(t\right)}(x)∥)\;\nabla u_{k}^{\left(t\right)}(x){)}_{m}$$

Here, c(∥∇u∥) is the conductivity function defined in Equation (25), which ensures the preservation of class boundaries by suppressing diffusion in high gradient regions. The subscript m indicates the application of the same anisotropic diffusion update at different spatial scales (core sizes).

(iv) Fixed Hyperparameters and Stability

In this study, the fractional order α, the gradient sensitivity parameter κ in the conductivity function, and the number of FPDE iterations T are treated as fixed hyperparameters rather than learnable parameters. These parameters are not updated through backpropagation. Specifically, α controls the degree of non-local diffusion, κ determines the edge sensitivity of the anisotropic conductivity function, and T defines the number of iterative refinement steps applied to the output probability maps. Only the network weights are learned during training, while the curriculum gate controls the contribution of the FPDE-refined probability maps in a gradual manner. To ensure numerical stability, T is kept limited, and the output values are clipped to the range [0, 1] after each update, as expressed in Equation (26).

(26) $$u_{k}^{\left(t+1\right)}\leftarrow \frac{u_{k}^{\left(t+1\right)}}{\sum_{k^{\prime }}u_{k^{\prime }}^{\left(t+1\right)}}$$

After each FPDE iteration, the class channels are renormalized pixel-wise, maintaining the condition $\sum_{k}u_{k}(x)=1$. This allows FPDERefine to act as a lightweight but effective modifier that enhances the spatial continuity of network outputs without causing over-smoothing.

#### 3.4.4. Curriculum Integration

Using the FPDERefine module at full efficiency at the beginning of training can lead to excessive smoothing and optimization instabilities on features that are not yet differentiated in early epochs. Therefore, the FPDERefine module is activated gradually using a curriculum learning approach. The aim is for the model to first learn basic class differentiation, and then for the spatial arrangement effect to be activated in a controlled manner.

In this context, FPDERefine is weighted on the probability maps Y^ generated from the main segmentation header with a gate function dependent on the epoch [48,49]. With the training epoch being e, the gate function g(e) is defined as in Equation (27).

(27) $$g(e)=\left\{\begin{array}{c} 0,\\ \frac{e-e_{\mathrm{start}}}{e_{\mathrm{ramp}}}\\ 1, \end{array}\right.,\begin{array}{cc} & e<e_{\mathrm{start}},\\ & e_{\mathrm{start}}\leq e<e_{\mathrm{start}}+e_{\mathrm{ramp}},\\ & e\geq e_{\mathrm{start}}+e_{\mathrm{ramp}} \end{array}$$

Here, $e_{\mathrm{start}}$ represents the epoch at which the FPDERefine module starts to activate, and $e_{\mathrm{ramp}}$ represents the transition period during which the module’s effect is linearly increased from zero to one. Thus, in the early phase, when g(e)=0, FPDERefine is completely deactivated; in the middle phase, its effect gradually increases; and in the late phase, it reaches full effectiveness. The gate function is directly integrated into the FPDERefine update. Accordingly, the diffusion update defined in Section 4.3 can be rewritten as in Equation (28).

(28) $$u_{k}^{\left(t+1\right)}=u_{k}^{\left(t\right)}-g(e)\;\lambda_{k}\;D_{\alpha }(u_{k}^{\left(t\right)}),\;k=1,\ldots ,K.$$

This formulation allows the FPDERefine module to function as a dynamic regulator throughout the training. In the early epochs, the model focuses on learning fundamental class differentiation via the UNet++ backbone and multi-head structure. In later epochs, FPDERefine refines the learned probability maps in terms of boundary continuity and regional consistency. In the architectural flow, FPDERefine is positioned after the softmax output of the main segmentation header and before the final argmax decision. This placement allows the module to operate directly in the probability space while maintaining differentiability and compatibility with multi-head learning. As a result, thanks to curriculum integration, FPDERefine takes on different roles at different stages of the training process. This increases both training stability and the final segmentation quality.

The FPDE formulation adopted in this study can be interpreted as a fractional extension of the classical Perona–Malik anisotropic diffusion model. In the standard Perona–Malik framework, image smoothing is guided by an edge-stopping diffusion coefficient that suppresses diffusion across strong gradients while promoting smoothing within homogeneous regions. In contrast, the proposed formulation replaces the integer-order diffusion operator with a fractional-order operator, allowing non-local interactions and long-range spatial dependencies to be incorporated into the refinement process. This enables the model to better preserve fine structures and discontinuities while reducing noise in the predicted probability maps.

In the proposed FPDE module, α was empirically set to 1.3 based on validation performance, whereas the number of FPDE iterations was set to T = 2 to balance refinement quality and computational efficiency. The diffusion coefficient is derived from the gradient magnitude of the prediction logits and is not directly learned as an independent parameter; instead, it is implicitly determined by the network outputs.

The gradient threshold parameter K, which controls the sensitivity of the diffusion process to edges, is also treated as a fixed scalar and selected empirically to balance edge preservation and noise suppression. Similarly, the number of FPDE iterations T is predefined (T = 2 in this study) to limit computational overhead while still providing effective refinement. Therefore, the FPDE module does not introduce additional learnable parameters, but rather acts as a physics-inspired post-processing layer integrated into the network in an end-to-end manner.

From an architectural perspective, the FPDE module operates directly on the output logits of the segmentation network and refines them through a small number of iterative updates. This design allows the refinement process to be seamlessly integrated into the training pipeline without significantly increasing the model complexity. By incorporating fractional-order diffusion, the module enhances spatial coherence and improves boundary delineation, particularly for minority classes such as rust disease regions, which tend to exhibit irregular and fragmented spatial patterns.

## 4. Training Strategy and Experimental Settings

This section outlines the training and evaluation procedures of the proposed model. The optimization strategies and loss functions employed for the multi-head network architecture are described in detail. The experimental configuration is also specified, including the division of the dataset into training and testing subsets, the selected patch sizes, training hyperparameters, and inference settings. In addition, the performance of the proposed approach is analyzed through comparisons with contemporary methods reported in the literature, as well as through comprehensive ablation studies. Both quantitative and qualitative criteria are used to assess the effectiveness of the model.

### 4.1. Training Strategy and Loss Functions

The proposed multi-head architecture is designed not only to improve segmentation accuracy but also to enhance the sharpness and spatial consistency of class boundaries. To achieve this goal, the model is optimized using a multi-task training strategy. During training, the network simultaneously learns the primary segmentation output together with two auxiliary components: an edge-aware head and a structurally guided Signed Distance Transform (SDT) head. This integrated learning framework enables the model to develop more precise and spatially consistent representations, both within class regions and along their boundaries.

#### 4.1.1. Multi-Head Loss Function

The total loss of the model is defined as the weighted sum of the main segmentation loss and the losses of the auxiliary headings and is given in Equation (29).

(29) $$L_{\mathrm{total}}=\lambda_{\mathrm{seg}}\;L_{\mathrm{seg}}+\lambda_{\mathrm{edge}}\;L_{\mathrm{edge}}+\lambda_{\mathrm{sdt}}\;L_{\mathrm{sdt}}$$

Here, $\lambda_{\mathrm{seg}},\;\lambda_{\mathrm{edge}},\;\lambda_{\mathrm{sdt}}$ are weighting coefficients that determine the relative contributions of segmentation, edge, and SDT headings, respectively.

#### 4.1.2. Main Segmentation Loss

The main segmentation heading is addressed as a multi-class pixel-level classification problem. In order to account for class imbalance and optimize both regional overlap and class separation, a combination of weighted cross-entropy and Dice loss is used. As shown in Equation (30).

(30) $$L_{\mathrm{seg}}=L_{\mathrm{WCE}}+L_{\mathrm{Dice}}$$

The weighted cross-entropy loss is given in Equation (31).

(31) LWCE=−1N∑i,j∑k=1Kwk Yi,j,klog(Y^i,j,k)

Here, $Y_{i,j,k}$ represents the ground-truth class label, Y^i,j,k represents the model output, and $w_{k}$ represents the weight of class k. The loss of dice directly maximizes regional overlap, as shown in Equation (32).

(32) LDice=1−2∑i,j,kYi,j,kY^i,j,k+ϵ∑i,j,kYi,j,k+∑i,j,kY^i,j,k+ϵ

This combined structure provides a balanced learning environment for both classes with large areas (e.g., soil, healthy vegetation) and those with more limited areas (low stress, high stress, rust disease).

#### 4.1.3. Edge-Sensitive Loss

The edge header was treated as a pixel-based binary classification problem [43]. Since the number of edge pixels is much smaller compared to the whole image, the binary cross-entropy given in Equation (33) was used for this header.

(33) Ledge=−1N∑i,j[Ei,jlog(E^i,j)+(1−Ei,j)log(1−E^i,j)]

Here, $E_{i,j}$ represents the ground-truth edge mask, and E^i,j represents the sigmoid output of the edge header. This loss encourages backbone representations to learn more distinctive features, especially at class boundaries.

#### 4.1.4. Signed Distance Transform (SDT) Loss

The SDT heading is defined as a regression problem that estimates the signed distance to the class boundaries [50]. The mean absolute error loss (L1) given in Equation (34) is used for this heading.

(34) Lsdt=1N∑i,j∣Di,j−D^i,j∣

Here, $D_{i,j}$ represents the normalized ground-truth SDT map, and D^i,j represents the SDT values predicted by the network. The loss of L1 prevents large errors from becoming overly dominant, offering a more stable and balanced learning process, especially in regions near class boundaries.

#### 4.1.5. Curriculum and Interaction with FPDE

In the curriculum learning-based integration described in Section 3.4.4, the FPDERefine module is not used directly as a loss term. Instead, it acts as an epoch-dependent output-space modifier on the main segmentation outputs. This structure allows the training process to proceed in two phases. In the early epochs, the loss functions focus on learning fundamental class distinctions. As training progresses, FPDERefine comes into play, refining the spatial consistency of the segmentation outputs. This decoupled optimization approach reduces the negative impact of multiple loss terms on each other and increases the overall stability of the optimization process. Pseudocode summarizing the general operation of the proposed method is presented in Algorithm 1.


**Algorithm 1. Pseudocode of proposed method.**


**Input**

*N training patches:* $I_{MS}:$ *multispectral patches, shape* (B×224×224×C) Y: *ground-truth masks,* K=5 *classes*

**Output**



Y^

*: final segmentation map*

**Auxiliary outputs**


E^

*: edge prediction*


D^

*: SDT prediction*

   **H variation includes Wavelet + Edge + SDT + FPDE + Curriculum + PatchBalance + BalancedMix**

**Step 1: Training loop**
1***for** epoch =* 1 *to* $N_{epochs}$
***do***

**Step 2: Batch construction (H-variation)**
2   ***for** each mini-batch* $\left(I_{MS}^{\left(b\right)},{\;Y}^{\left(b\right)}\right)$ ***do***3      
Apply PatchBalance to adjust patch−level sampling.
4      Construct batch stream using BalancedMix (water/rust).

**Step 3: Auxiliary ground-truth preparation**
5      **for** each mask $Y_{i}\in Y^{\left(b\right)}$
**do**6          *Compute edge ground truth* $E_{i}^{gt}$ *via morphological gradient. ←* Equation (15)7          *Merge multi-class boundaries into a single edge representation (if required). ←* Equation (16)8          *Compute signed distance transform* $D_{i}^{gt},$ *←* Equation (17)9          *Scale and clip SDT values for numerical stability. ←* Equation (19)10      
***end for***


**Step 4: Forward pass (UNet++-H backbone)**
11      
*Pass* $I_{MS}^{\left(b\right)}$ *through UNet++ encoder–decoder.*
12      *Build dense skip connections and obtain fused feature map* $x^{0.4}.$13      *Apply wavelet-enhanced feature processing within convolutional blocks (H enabled).*


**Step 5: Multi-head prediction**
14      Compute segmentation logits Z from $x^{0,4}.$ ← Equation (12)15      Predict edge map E^(b) via edge head. ← Equations (13) and (14)16      Predict SDT map D^(b) via SDT head. ← Equation (18)

**Step 6: FPDE-based output refinement (H-variation)**
17      Convert logits to probability maps $P^{\left(0\right)}.$ ← Equation (7)
      Initialize $u_{k}^{\left(0\right)}\leftarrow P_{k}^{\left(0\right)}$ for all classes k. ← Equation (24)18      for t=1 $\mathrm{to}\;T_{FPDE}$
**do**19         Compute edge-stopping conductivity from local gradients. ← Equation (22)20         Update $u_{k}^{\left(t+1\right)}$ using anisotropic multi-scale FPDE. ← Equation (25)21         Clip $u_{k}^{\left(t+1\right)}$ *to* [0, 1] for numerical stability. ← Equation (26)22         Renormalize per pixel: $u_{k}^{\left(t+1\right)}\leftarrow \frac{u_{k}^{\left(t+1\right)}}{\sum_{k^{\prime }}u_{k^{\prime }}^{\left(t+1\right)}}$
23      **end for**

**Step 7: Curriculum gating for FPDE**
24      Compute curriculum gate g(epoch) ← Equation (27)25      Blend refined and original probabilities using g(epoch) ← Equation (28)26      Obtain final segmentation probabilities P^(b).


**Step 8: Loss computation (multi-task)**
27      Compute segmentation loss $L_{seg}.$ ← Equations (30)–(32)28      Compute edge loss $L_{edge}$ ← Equation (33)29      Compute SDT loss $L_{sdt}.$ ← Equation (34)30      Compute total loss ← Equation (29)

**Step 9: Optimization**
31      Backpropagate $L_{total}$ and update network parameters (Adam).32   **end for** (mini-batch loop)33**end for** (epoch loop)

**Step 10: Inference**
34   Compute P^ (with FPDE refinement if enabled).35   Output final prediction

Algorithm 1 outlines the training and inference processes for the H variation of the proposed method. During the training phase, balanced mini-batches are generated using PatchBalance and BalancedMix strategies to ensure balanced sampling from multiple stressors. Edge maps and Signed Distance Transform (SDT) lines are also automatically generated as auxiliary control signals. The WF-UNet++-H backbone performs wavelet-assisted multi-scale feature extraction. Multi-tasking learning is conducted with the main segmentation header in addition to the Edge and SDT auxiliary heads. Class probability maps obtained from the main header are refined by an FPDE-based refinement module operating in the output space. This module is controlled by a curriculum gate that is gradually activated throughout training, in accordance with curriculum learning. In the inference phase, only a five-class final segmentation output refined with FPDE is produced. Auxiliary heads are not used in this phase.

### 4.2. Experimental Settings

In this study, all experiments were structured to ensure a reproducible and fair evaluation of the proposed architecture. All models were trained with the same hardware, software environment, data splitting, and training settings. Experiments were conducted on a workstation with an NVIDIA RTX 4070 GPU (8 GB VRAM), an Intel-based multi-core CPU, and 32 GB of RAM. Python 3.x and TensorFlow 2.10 were used in the software environment, and dynamic memory allocation was enabled to prevent GPU memory overflows. In order to minimize the effects of stochastic variation, the value of the random seed was set constant at 42 for all the experiments. The training process for the models was performed using 224 × 224 pixel images in batches of 4, and each model was trained for a maximum of 80 epochs. Validation metrics were calculated after the completion of each epoch, and the weights for the validation score were saved for the epoch with the highest validation score. There was no use of early stopping; the selection of the model was based only on validation performance.

The Adam optimization algorithm was used with an initial learning rate of 0.002. The learning rate schedule was based on the ‘Reduce on Plateau.’ The learning rate was reduced by a factor of 0.5 every time the validation loss did not improve for 10 epochs. The reduction continued until it reached a minimum value of 1 × 10^−6^. There was no weight decay or gradient clipping used during training. Data augmentation was also not used, and the network was trained on the original image patches.

The data was then randomized at the patch level for the training and validation sets, ensuring that patches representing both water stress and rust disease were included in the evaluation. The class imbalance was reduced indirectly through class weights and patch selection. The output of the model was a single-band image representing the segmentation mask. Since the orthomosaics were divided into overlapping patches using a stride smaller than the patch size, spatial correlation may exist between neighboring patches assigned to different subsets. Therefore, the reported validation results should be interpreted as patch-level within-dataset performance rather than fully independent spatial generalization. This limitation is further discussed in Section 6, and future work will adopt spatially disjoint block-wise and leave-one-field-out validation protocols.

In terms of quantitative evaluation, the class-wise IoU and mean IoU were considered as the primary metrics. Qualitative evaluation was also performed by comparing the model predictions with the ground-truth mask in the scenes. This experimental setup enables the evaluation of the model in terms of training stability and performance when the multi-head loss structure and the FPDERefine are jointly optimized.

### 4.3. Inference Strategy and Evaluation Metrics

During the inference process, the high-resolution orthomosaic images were processed using the sliding window approach with the patch size being the same as the one used during the training process, which is 224 × 224. For the removal of blocking effects at the class boundaries and to improve the spatial continuity, the overlapping inference process was utilized with a stride of 96 pixels.

In accordance with this inference, class probability maps based on overlapping regions were blended by averaging multiple predictions related to the same pixel, as provided by soft blending. In the final step, class labeling was carried out by applying the argmax operation to the probability distribution. The aforementioned procedure of inference enabled smooth, continuous, and structurally coherent reconstruction of large areas of orthomosaic regions.

Quantitative metrics for model performance evaluation are based on the conventional metrics for multi-class semantic segmentation. The intersection over union metric was calculated for each class, and the mean IoU was considered the primary metric for model performance evaluation. The Dice coefficient was also calculated and reported as a supplement to the IoU metric for a more sensitive evaluation of the quality of the region overlap. As the stress and disease-affected areas in the agricultural images are likely to be small in size, the average metrics may not be sufficient for the evaluation. Therefore, the Foreground IoU was also considered as a metric for model evaluation. This metric was calculated by ignoring the background classes. This metric is considered a more representative evaluation metric for the model performance, particularly for the small but critical classes, i.e., low stress, high stress, and rust disease.

## 5. Experimental Results

This section presents the experimental performance of the proposed multi-head UNet++-based WF-UNet++ semantic segmentation framework. This includes quantitative comparisons, detailed class-wise analysis, visual examples of scenes of interest, and ablation study of the important architectural elements of the proposed framework. All the experiments were conducted with the experimental setup and strategy discussed in Section 4. These experiments provide a complete analysis of the overall performance of the proposed framework as well as the individual contributions of its various elements. All experiments are conducted within the available dataset setting

### 5.1. Comparison with State-of-the-Art Methods

Quantitative evaluation of the proposed method was performed through comparison with the state-of-the-art convolutional neural network (CNN) and transformer-based semantic segmentation architectures. The comparison was performed using the overall performance metrics corresponding to the best validation epochs and the class-wise pixel-level F1 score evaluation metrics. The results are tabulated in Table 2 and Table 3, respectively. In order to ensure fairness in the comparison, the proposed method and the state-of-the-art architectures were trained and tested using the same data split with an input resolution of 224 × 224 pixels and a unified evaluation metric. This design allows a fair evaluation of the quantitative improvements achieved using the proposed method in an unbiased manner.

The results shown in Table 2 and Table 3 clearly indicate that our method always achieves higher performance than existing architectures on all metrics of segmentation accuracy and per-class pixel prediction performance. The performance of our model on the validation dataset was 0.8623 mIoU, 0.8439 FG-IoU, and 0.9250 Dice, which indicates that our method has the best performance compared to all existing architectures. In comparison to existing state-of-the-art architectures based on CNNs, such as UNet and DeepLabV3+, our method has been shown to achieve improvements of around 6.8–7.7% on mIoU and 4.1–4.7% on Dice.

In the class-wise F1 score table given in Table 3, the balanced performance across all classes is evident. Here, the F1 score values of 0.9210, 0.9430, and 0.8610 were obtained for the low-stress, high-stress, and rust disease classes, respectively. Also, the proposed architecture, despite having a relatively low number of parameters, i.e., 6.54 million, performs better than some transformer-based architectures with a significantly higher number of parameters. This demonstrates the efficient trade-off between computational efficiency and segmentation performance.

In summary, the above findings validate that the proposed method indeed offers a sound solution for the task of semantic segmentation, as indicated by the overall as well as specific class-based performance and generalization.

### 5.2. Class-Wise Quantitative Results (Five-Class IoU Analysis)

To further test the performance of the proposed approach for the five-class semantic segmentation task, the class-wise IoU values were calculated for the validation set. The results, as shown in Table 4, give a quantitative idea about the performance of the model for discriminating between classes. To show the performance of the model for the overall segmentation task, the overall performance metrics such as mIoU, FG-IoU, and Dice coefficient values are provided in Table 5. Here, the objective is to reveal not only the performance of the model for the overall task but also the performance for discriminating between classes, as there might be a class imbalance.

The results on a per-class basis are provided in Table 4, which shows that the best performance was obtained by the soil class with an IoU score of 0.9359. This can be explained by the large coverage area of this class and its homogeneous structure. The healthy vegetation class was also learned effectively, with an IoU score of 0.8924, showing that it was learned reliably and distinguished from stressed regions. The low-stress and high-stress classes were learned with IoU values of 0.8755 and 0.8522, respectively, which show that spectrally similar but physiologically different stressed regions can be learned gradually and separately. It is also important to notice that reliable performance on the high-stress class shows that severely stressed regions can be learned with clear and distinguishable boundaries.

For the rust disease class, which has limited spatial extent and heterogeneous texture, it was found that the IoU value was 0.7554, which indicates that this was indeed a more challenging segmentation task. However, it also indicates that there was discrimination between these areas of infection. If all classes are considered as a whole, it was found that the mIoU value was 0.8623, which indicates that all classes were balanced, regardless of whether they were dominant or not. As expected, based on the global metrics provided in Table 5, it was found that the FG IoU and Dice values were 0.8439 and 0.9250, respectively, which indicates that agriculturally important foreground classes were segmented with high accuracy.

Overall, the combined class-level and global evaluations confirm that the proposed approach provides a stable, generalizable, and reliable semantic segmentation solution under varying field conditions.

### 5.3. Optimization Behavior and Class-Wise Performance

**Figure 7:**
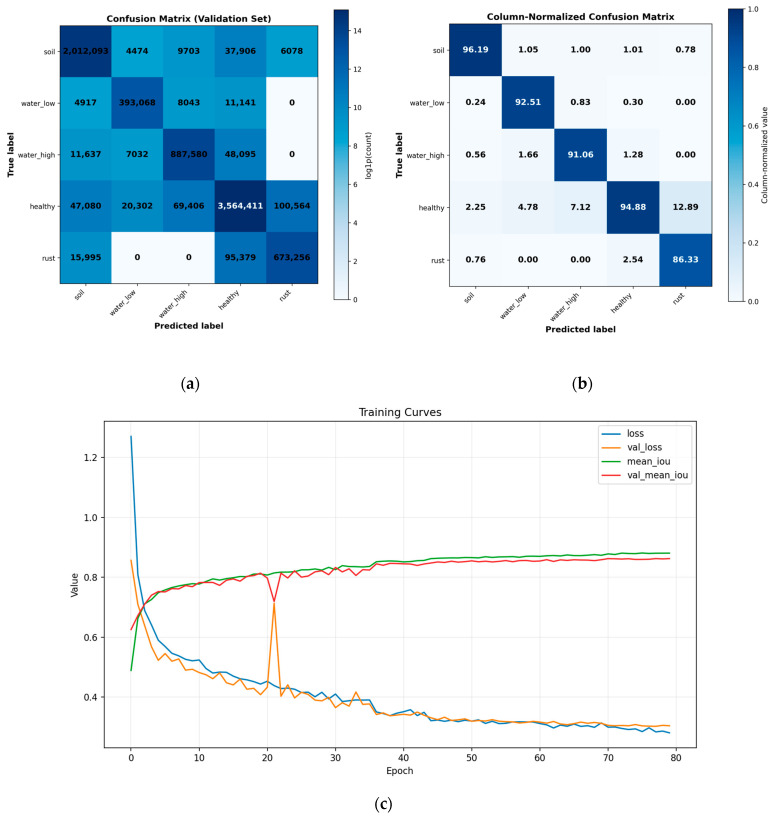
Comprehensive performance analysis of the proposed UNet++-FPDE model, including (**a**) raw confusion matrix, (**b**) column-normalized confusion matrix, and (**c**) training and validation loss together with mean IoU evolution across epochs.

Figure 7 describes the optimization stability and class-wise prediction performance of the proposed UNet++-FPDE (WF-UNet++) architecture during the training process. It can be seen that the training and validation curves demonstrate regular convergence. This indicates that there is no overfitting or bias in the model. Moreover, the confusion matrix obtained during the validation process indicates high diagonal dominance. This indicates that the model can differentiate between the various classes. There is moderate interference between low-stress and high-stress classes. This can be attributed to the physiological and spectral similarities of these two classes. This also indicates that the model can differentiate between the two levels of stress. There is less interference between the rust disease class and the healthy vegetation class. This indicates that the model can precisely locate the boundaries. Moreover, the rust disease class is also identified.

Figure 7 demonstrates the assessment of the learning dynamics and class-wise prediction performance of the proposed framework. Figure 7a presents the raw confusion matrix, where the strong diagonal dominance indicates accurate discrimination among soil, healthy vegetation, low stress, high stress, and rust disease classes. Figure 7b shows the column-normalized confusion matrix, further confirming that the model maintains high classification consistency across the five classes. A limited confusion is observed between the low-stress and high-stress classes, which can be attributed to their physiological continuity and spectral similarity. In contrast, the very low misclassification rate between rust disease and healthy vegetation indicates the boundary localization capability of the proposed framework, supported by the edge-aware components and SDT-based structural guidance. Figure 7c illustrates the training and validation loss curves together with the mean IoU evolution across epochs. The stable convergence behavior and gradual increase in mean IoU demonstrate that the curriculum-based FPDERefine module contributes to stable optimization and improved discriminative feature learning.

On the whole, the joint analysis of convergence dynamics and the accuracy of the two classes points to the stable and continuous nature of the optimization process. Moreover, the results clearly prove the high discriminative capability of the model in the context of multi-class stress segmentation. In conclusion, these findings provide strong qualitative support to the quantitative results presented in Section 5.

### 5.4. Qualitative Results (Patch-Level Visualization)

In this section, the visual performance of the proposed method at the patch level will be evaluated based on representative examples. The visual evaluation aims to evaluate the observable counterparts of the class-based quantitative evaluation. Additionally, the performance of the model at the class boundaries, transition regions of stress levels, and small-scale rust disease regions will be further explored. All the examples used in the evaluation will be based on the validation set, and the prediction of the model will be compared with the spatial accuracy masks.

**Figure 8:**
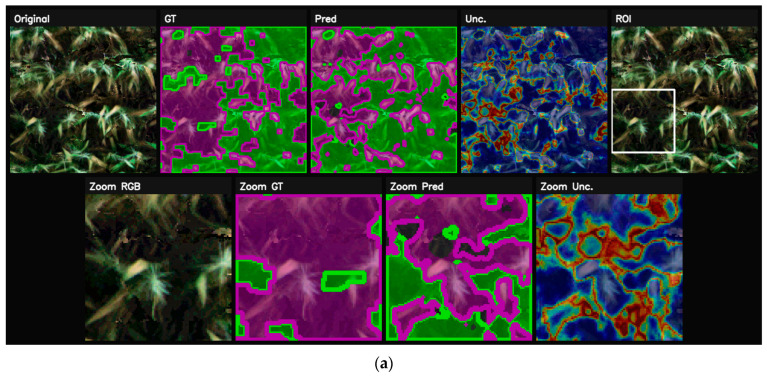

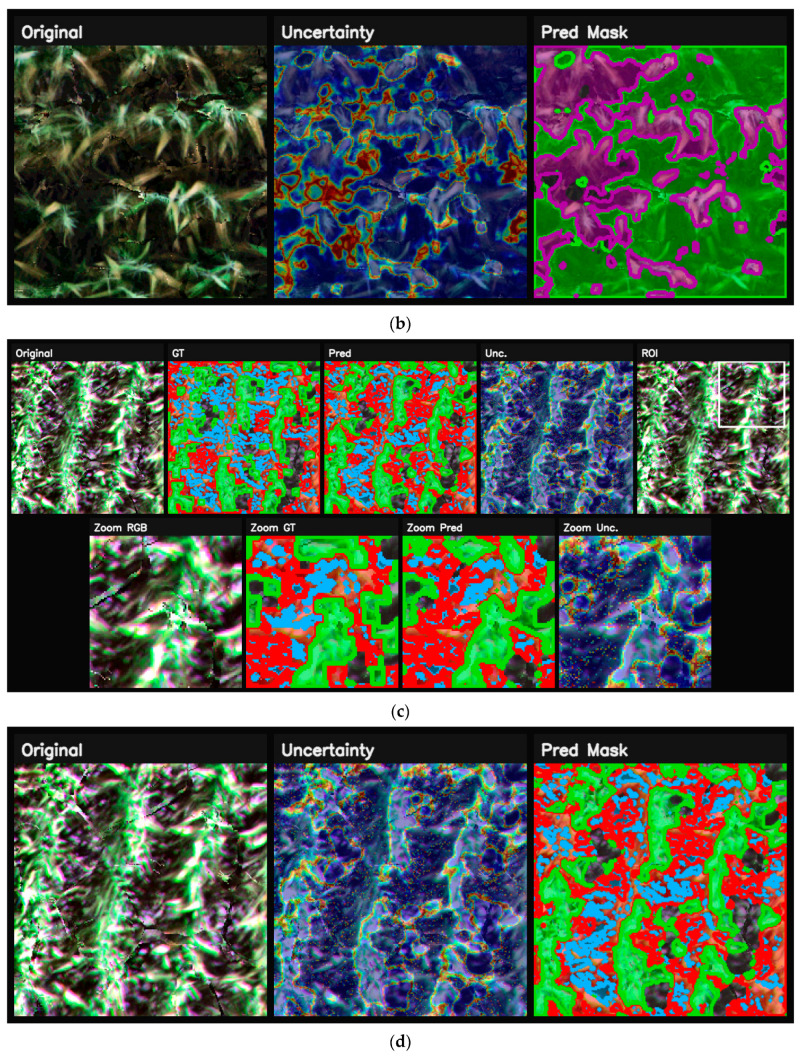
Patch-level qualitative segmentation results and uncertainty visualization for rust disease and water stress scenarios. (**a**,**b**) Representative patch-level examples illustrating rust disease segmentation, including the original RGB image, ground truth labels, predicted masks, and corresponding uncertainty maps. (**c**,**d**) Representative patch-level examples illustrating water stress segmentation under heterogeneous field conditions, together with the associated uncertainty visualizations.

Figure 8 shows a set of patch-level visual exemplars of the segmentation performance of the proposed model under rust disease stress and water stress conditions, along with the corresponding uncertainty distribution. From the rust disease samples in Figure 8a,b, it can be observed that the spatial extent of the disease stress regions is small, yet there is a high degree of concordance in the model prediction with the spatial accuracy masks.

In the water stress examples depicted in Figure 8c,d, boundary regions do not maintain their distinctness, particularly within the transition regions used to separate the low and high-stress areas. Such behavior was expected based on the spectral similarity of these two classes and the smooth physiological transition of stress. The model maintains high spatial continuity and discrimination capability for the soil and healthy vegetation classes.

The analysis of the maps indicates that areas of higher uncertainty values are mostly located along class boundaries, transition areas, and small disease patches. The indication that the model may be inadequate is, therefore, rather a result of intended confidence estimation that highlights areas that are inherently uncertain or difficult to classify. Figure 8 effectively identifies areas where model predictions are reliable and areas that may require additional validation, thereby providing a significant advantage to decision processes used in precision agriculture.

### 5.5. Orthomosaic-Level Results

In this segment, the output of the segmentation at the orthomosaic scale, resulting from the aggregation of the patch-level prediction, will be presented. The aim of the analysis here is to not only evaluate the accuracy of the model at a local level but also to examine the spatial continuity, inter-class consistency, and the applicability of the method for extensive field installations. All the outputs have been achieved by the application of overlapping patch extraction and blending strategies, which have reduced the blocking effects of the model.

**Figure 9:**
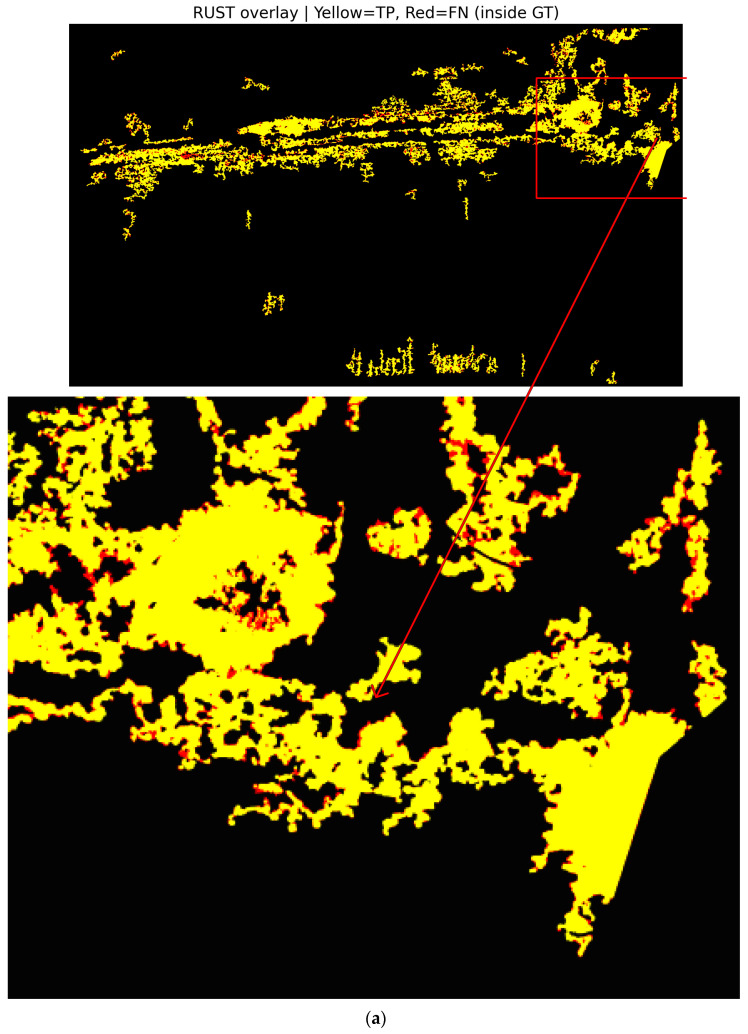

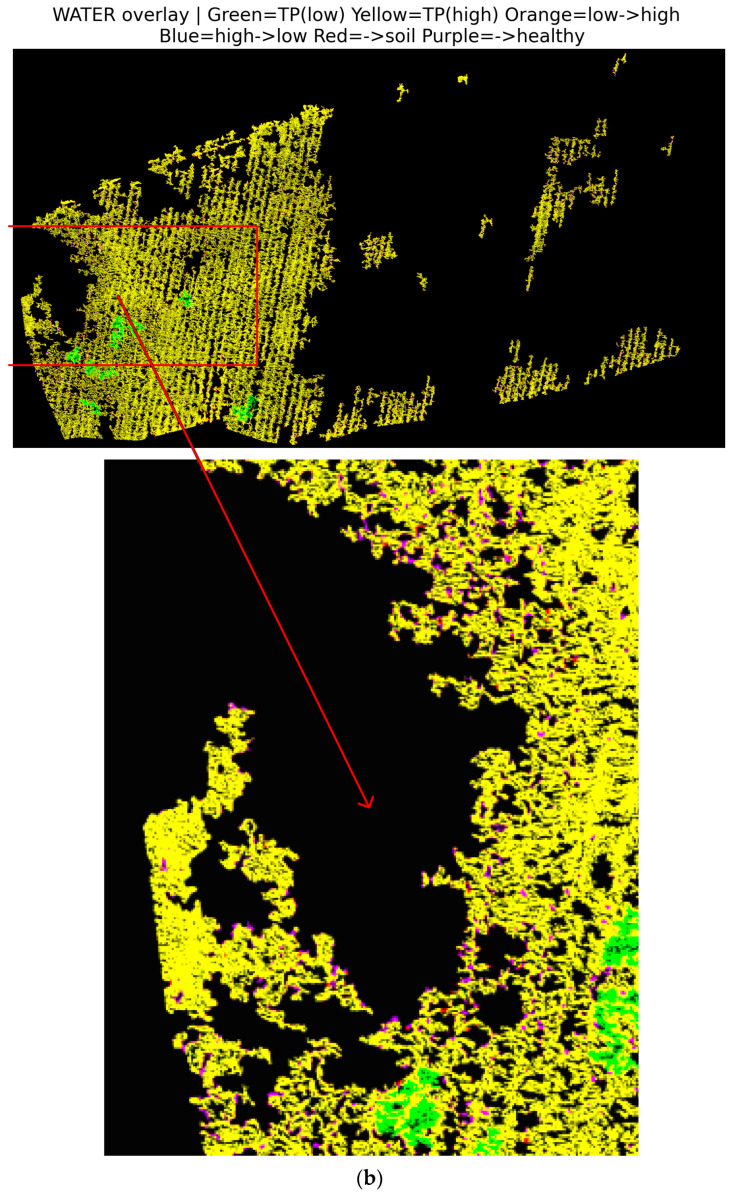
Illustrates the orthomosaic-level segmentation outputs of the proposed method. (**a**) Rust disease overlay, where yellow indicates true positives detections and red denotes false negatives within ground truth regions. (**b**) Water stress overlay, highlighting correct low-stress and high-stress predictions as well as class transition errors between stress levels and other land cover classes. Red rectangles indicate regions of interest (ROI), with magnified views provided to emphasize local segmentation behavior and error patterns.

Figure 9 presents the orthomosaic-level segmentation outputs obtained by projecting patch-level predictions back onto the original field scale. Figure 9a shows the rust-disease overlay, where true-positive detections are mainly organized as spatially continuous and interconnected structures along plant rows. The false-negative regions are mostly located in narrow and fragmented zones close to class boundaries, indicating that the remaining errors are concentrated in difficult transition areas rather than being randomly distributed across the field. This result demonstrates that the proposed method can preserve the spatial structure of localized rust infection regions at the orthomosaic scale. Figure 9b presents the water stress overlay, including both low-stress and high-stress regions. The results show that water stress patterns are segmented consistently over large field areas, while most errors occur around the transition zones between low and high stress. These errors are expected because the two stress levels represent a gradual physiological continuum and exhibit spectral similarity. Overall, Figure 9 confirms that the proposed overlapping patch-based inference strategy can be reliably scaled from patch-level prediction to orthomosaic-level field mapping while maintaining spatial continuity and class consistency.

### 5.6. Ablation Study

This section presents a comprehensive ablation study designed to investigate the contribution of individual components within the proposed architecture to overall model performance. The experiments systematically evaluate the quantitative impact of progressively introduced architectural and training elements, beginning with the baseline configuration (Variation A) and extending to the validation set. In particular, the effects of the FPDE-based optimization module, the contributions of the edge-aware and Signed Distance Transform (SDT) components, and the rationale underlying the selection of the final Variation H are examined in detail.

Table 6 demonstrates the ablation variants A through H, indicating the architectural components being activated. Beginning with the baseline (Variation A), the study gradually incorporates wavelet-based feature enrichment, edge-aware and SDT-guided supervision, curriculum learning-assisted FPDE refinement, and class balancing. This iterative design allows for the evaluation of the individual and combined contributions of each component towards the overall performance.

Although the proposed WF-UNet++ framework exhibits a longer training time compared to simpler baseline models such as UNet, this increase is primarily attributed to the integration of the FPDE-based refinement module and multi-head supervision strategy. It is important to note that this additional computational cost is mainly incurred during training, whereas the impact on inference time remains relatively limited due to the small number of FPDE iterations (T = 2) and the lightweight nature of the refinement process. From a practical perspective, the proposed method achieves a favorable trade-off between computational complexity and segmentation performance. The observed improvements in boundary delineation, spatial coherence, and minority-class detection, particularly for rust disease regions, justify the additional training overhead. Moreover, in UAV-based precision agriculture applications, model training is typically performed offline, while inference is deployed in field conditions. In this context, the proposed framework may be suitable for offline or near-real-time field-scale analysis after dedicated inference-time benchmarking, since the refinement module uses only a small number of iterations. However, explicit onboard UAV real-time deployment was not evaluated in this study. Therefore, the proposed method is not intended to minimize computational cost alone, but rather to achieve a balanced optimization between accuracy, robustness, and practical applicability in real-world agricultural monitoring scenarios.

The improvements observed in Table 7 are also reflected at the individual class level. Table 8 provides the class-wise F1 performance of the ablation variants and confirms this trend. Compared with the baseline Variation A, the final Variation H achieves higher F1 scores for all five classes. The rust disease class increases from 0.8208 to 0.8610, while the low-stress and high-stress classes increase from 0.8682 to 0.9210 and from 0.9207 to 0.9430, respectively. These improvements are particularly important because low stress, high stress, and rust disease correspond to the agriculturally critical and relatively more challenging foreground classes.

The class-wise results indicate that the proposed components improve not only the global segmentation performance but also the recognition of small, heterogeneous, and imbalanced stress-related regions. Therefore, the overall improvements reported in Table 7 are supported by the complementary evidence provided by the class-wise F1 results in Table 8. The edge-aware and SDT-based auxiliary branches contribute mainly to boundary delineation and local structural consistency, whereas the FPDE module improves spatial coherence in fragmented regions. In addition, the use of curriculum learning and balancing strategies stabilizes the learning process and improves the consistency of the model across classes.

#### 5.6.1. Comparison of A → H Variations

At this stage, the improvement in performance resulting from the incremental addition of the proposed UNet++ architecture was evaluated systematically, commencing with the basic variant (Variation A). The term “Variation A” refers to the basic architecture, which consists only of the primary segmentation head and the conventional loss formulation. In the next variations, the additional components were added incrementally: edge-aware supervision, Signed Distance Transform (SDT) geometric representation, FPDE output refinement, integration with curriculum learning, and balancing at the patch level.

The incremental extension in the architecture facilitates the isolated and systematic evaluation of the contribution of each module to the validation performance. As can be seen in Table 7, the base performance of Variation A, with metrics of 0.7971 mIoU, 0.7707 FG-IoU, and 0.8853 Dice, increases to 0.8623 mIoU, 0.8439 FG-IoU, and 0.9250 Dice in the final Variation H, as a result of the progressive addition of the various components of the architecture. The increases in the metrics of the base model by 6.8–7.7 percent in mIoU and 4.1–4.7 percent in Dice indicate that each of the components of the architecture makes a positive contribution to the performance. The significant improvement in the performance of the models in Variation G and H indicates that the use of both the FPDE-based refinement and balancing strategies significantly improves the consistency of the segmentation.

The class-specific F1 scores presented in Table 8 also support the same trend. In other words, the F1 score of the rust diseases increases from 0.8208 to 0.8610 in Variation H compared to Variation A. In addition, the low-stress and high-stress classes also demonstrate improvements in their F1 scores from 0.8682 to 0.9210 and from 0.9207 to 0.9430, respectively. This indicates that the edge-aware supervision and SDT-based geometric guidance help sharpen the class boundaries. At the same time, the FPDE module helps to improve the spatial consistency, especially in heterogeneous and fragmented regions. Moreover, the use of balancing strategies with curriculum learning helps to improve the stability of the training process. As a result, the performance of the model is more consistent across the classes.

The architecture that has been proposed, based on the modular and multi-head design, demonstrates a progressive improvement in the accuracy of semantic segmentation. Each module makes a significant contribution to the overall performance metrics and discrimination power.

#### 5.6.2. Impact of the FPDE Module (ON/OFF Analysis)

The effect of the fractional-order anisotropic diffusion-based output refinement module (FPDE) is also validated by comparing architectural variations of the model with the module enabled versus disabled. When the FPDE module is not used, the class boundary appears to be blurred, predictions in the rust disease area are fragmented, and changes between low- and high-stress areas are not smooth, indicating that the model is not able to maintain its ability to preserve coherence in the scene.

Quantitative validation of the effect of the FPDE module is provided in Table 7, which shows that while the mIoU remains close to 0.81 in all cases in which the FPDE module is not used, the mIoU increases to 0.8421 in Variation G and 0.8623 in Variation H, in which the FPDE module is used along with the corresponding compensation techniques. Similarly, the FG IoU increases to 0.8439, while the mean Dice score increases to 0.9250, compared to 0.8214 and 0.9131, respectively, in the cases in which the FPDE module is not used. Table 8 provides the F1 score of each class, which increases to 0.8610 in the rust class, while the low-stress class increases to 0.9210, and the high-stress class increases to 0.9430, compared to 0.8682, 0.9207, respectively, in the cases in which the FPDE module is not used.

These results demonstrate that the FPDE module should not be considered as a simple post-processing step for image smoothing. Rather, it should be considered as a key component which works in close integration with the learning process, in order to improve the spatial consistency and the quality of the segmentation, especially in the presence of heterogeneous class structures.

#### 5.6.3. Contribution of Edge and SDT Headers

In this part of the ablation study, the impact of the edge-aware head and the Signed Distance Transform head on the performance of the segmentation model was investigated. Architectural variations of the model without these two types of heads showed increased class leakage and boundary ambiguity, particularly on slender class boundaries. The edge-aware head provides sharper class boundary delineation, while the SDT head enables more consistent class geometry learning.

The combined integration of these two auxiliary heads provided complementary benefits, particularly in boundary delineation and structural regularity for heterogeneous stress patterns and fragmented rust disease regions. However, their effect on aggregate metrics was less dominant than that of the FPDE refinement and class-balancing components. Therefore, the edge-aware and SDT heads should be interpreted as supporting refinement mechanisms that improve spatial coherence and boundary quality rather than as the primary drivers of overall performance improvement.

#### 5.6.4. Rationale for Choosing the H Variation

When comparing the ablation study outcomes in general, Variation H is found to be the most balanced in terms of enhancement in performance and architectural complexity. As shown in Table 7, Variation H achieves the best overall quantitative performance among all ablation variants, with the highest mIoU, FG-IoU, and mean Dice scores. This result shows that the strongest performance is obtained when FPDE-based output refinement, edge-aware supervision, SDT-based geometric guidance, curriculum learning, PatchBalance, and BalancedMix are integrated into a single configuration. The corresponding class-wise F1 scores in Table 8 further confirm that Variation H provides the most balanced performance across all classes, including the minority and agriculturally critical foreground classes. Therefore, Variation H was selected as the final proposed configuration, and all subsequent experimental analyses were conducted using this variant.

### 5.7. Geospatial Localization of Rust-Affected Areas

In this section, it is shown that the proposed method not only performs pixel-wise semantic segmentation but also carries out field-scale spatial localization of the rust-affected areas. This is because the predictions obtained at the patch level from the model are reprojected back to the original orthomosaic coordinate system, thereby allowing the infected areas to be represented not only as classified pixels but also as spatially referenced entities. This would allow the geographic mapping of the disease distribution and the evaluation of various spatial attributes, such as the patterns of spread, boundaries, and area.

Consequently, the output of the model provides operationally relevant information that can be used in decision support systems. In this context, it can be said that the study contributes to the current literature beyond image-based classification since it introduces a spatial decision support framework that can be used in precision farming.

**Figure 10:**
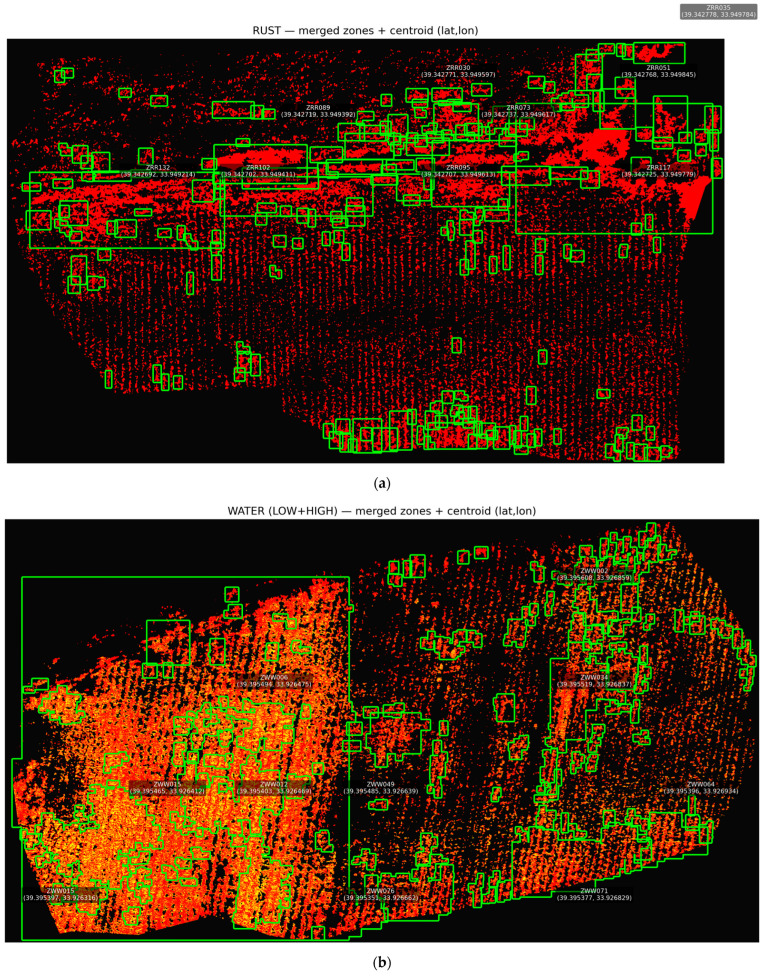
Field-scale visualization of merged stress zones derived from model predictions. (**a**) Rust disease merged zones. (**b**) Water stress merged zones including both low and high stress regions. Predicted stress regions are shown in red/yellow, while merged zones are outlined in green to improve visual contrast and clearly highlight spatial grouping of detected regions. Only the most representative zone centroids are annotated for clarity.

Figure 10 presents the field-scale spatial aggregation of predicted stress regions into merged zones. As observed, the proposed merging strategy effectively groups fragmented pixel-level predictions into coherent spatial regions, improving interpretability at the field scale. Water stress regions (Figure 10)) exhibit more continuous and widespread patterns, whereas rust disease regions (Figure 10b) appear more localized and clustered. The use of green bounding zones enhances visual clarity and allows for a clearer distinction between individual stress clusters and the underlying prediction map. This representation demonstrates the practical applicability of the proposed framework for large-scale UAV-based agricultural monitoring.

To further validate the results obtained through the graphical representation, a spatial summary of the predicted rust zones obtained through the component analysis has been presented in the form of Table 9. In the table, the location of the identified zones of rust diseases has been presented along with the size of the area. This provides a quantitative representation of the field-wise distribution of the rust diseases. At the same time, the spatial summary of the identified zones of water stress has been presented in the form of Table 10. In the table, the location of the identified low and high water stress zones has been presented. As shown in Table 9 and Table 10 presented above reveals that the results obtained through the segmentation provide information that can be used to determine the intensity of the diseases and the extent of the water stress. Thus, the proposed method can be effectively used to determine the diseases and the extent of the water stress.

## 6. Discussion

It is important to clarify that the proposed framework does not aim to detect multiple coexisting stress conditions within a single field scene. Instead, this study focuses on the semantic unification of heterogeneous UAV multispectral datasets representing different stress conditions (water stress and rust disease). Therefore, the contribution lies in establishing a shared multi-class segmentation space across distinct datasets with different annotation schemes, rather than modeling simultaneous multi-stress interactions within the same spatial context. This distinction is critical for correctly interpreting the scope and contribution of the proposed approach.

While the dataset used in this study is limited to two UAV multispectral orthomosaics acquired from maize fields within the same geographical region, the primary objective of this work is not to claim broad generalization across diverse agricultural conditions. Instead, the study is designed as a proof-of-concept to demonstrate the feasibility of a unified semantic segmentation framework for integrating heterogeneous stress conditions into a common label space. Therefore, the proposed approach should be interpreted as a methodological contribution focusing on dataset harmonization and physics-informed refinement, rather than a fully generalized solution applicable to all crop types and environments.

A limitation of this study is that the datasets were collected from two maize production fields located in the same geographical region (Kırşehir, Turkey). Although this allows for controlled analysis of both water stress and rust disease conditions, it may restrict the generalization of the proposed model to different climatic regions, crop varieties, or growth stages. However, it is important to note that the proposed framework is not inherently dependent on a specific dataset. The use of multispectral inputs and physically interpretable vegetation indices (e.g., NDVI, NDRE, SAVI) provides a generalizable representation of plant health conditions, which can potentially be transferred to different environments. Moreover, the FPDE-based refinement module operates at the output level and is independent of the specific spectral characteristics of the training data, further supporting its adaptability. Another limitation arises from the ground-truth generation process, which relies on index-based thresholding. While this approach enables large-scale label generation without manual annotation, it may introduce inaccuracies that can propagate into the training process. Nevertheless, the UAV data acquisition was performed using an RTK-enabled platform, providing high spatial and geometric accuracy in the orthomosaic generation process. This high positional precision helps ensure that spectral measurements are spatially consistent and reduces potential misalignment errors in the derived ground-truth masks. In addition, the use of multiple spectral indices and post-processing steps further mitigates potential labeling errors and provides a consistent approximation of stress regions.

Another important aspect concerns the reliability of the ground-truth labels. In this study, the labels are generated using a rule-based approach derived from multiple vegetation indices, including NDVI, NDRE, SAVI, and spectral ratio metrics. While this approach does not rely on manual expert annotation, it is grounded in well-established physical and physiological relationships between spectral responses and plant stress conditions. To improve label consistency, multiple indices are jointly evaluated using thresholding and decision rules, and spatial post-processing steps such as morphological filtering are applied to reduce noise and eliminate isolated artifacts. This multi-criteria strategy helps mitigate the limitations of single-index thresholding and provides a more stable approximation of stress regions. Furthermore, the UAV data acquisition was conducted using an RTK-enabled platform, ensuring high spatial and geometric accuracy in the orthomosaic generation process. This reduces potential spatial misalignment between spectral measurements and derived labels, thereby improving the reliability of the generated ground truth. Although the labeling process is not based on manual annotation, it provides a scalable and physically interpretable alternative for large-scale UAV-based agricultural analysis. Nevertheless, we acknowledge that rule-based labeling may still introduce uncertainties. Future work will focus on incorporating manually annotated samples and hybrid labeling strategies to further validate and refine the ground-truth generation process.

We acknowledge that the train–validation split in this study was performed at the patch level. Due to the overlapping patch extraction strategy (stride < patch size), spatially adjacent or partially overlapping regions may be present in both the training and validation sets. This can introduce spatial correlation between the two sets and may lead to optimistic performance estimates. Therefore, the reported results should be interpreted as patch-level within-dataset validation performance rather than as evidence of fully independent cross-field, cross-region, or cross-season generalization. This limitation is particularly important for UAV orthomosaic data, where spatial autocorrelation can be strong between neighboring patches. Future work will adopt spatially disjoint block-wise validation and leave-one-field-out testing protocols to provide a more rigorous assessment of model generalization under independent field conditions.

The contribution of the edge-aware and SDT auxiliary branches should be interpreted with caution. While these components do not consistently yield large improvements in overall quantitative metrics across all experiments, they provide complementary benefits in specific aspects of the segmentation task. In particular, the edge-aware branch contributes to sharper boundary delineation, while the SDT branch encourages structural consistency in spatial predictions. These effects are more evident in qualitative visualizations and class-specific regions rather than in aggregated performance metrics such as mIoU. Therefore, the role of these auxiliary heads is better understood as supporting refinement mechanisms that enhance spatial coherence, rather than as primary drivers of performance improvement.

The generalization ability of the proposed model has not been extensively evaluated beyond the current dataset setting. All experiments in this study are conducted on UAV multispectral data collected from a limited number of field scenes, and no cross-region, cross-season, or cross-crop validation is performed. Therefore, the reported results should be interpreted within the current dataset setting and should not be considered evidence of broad cross-region, cross-season, or cross-crop generalization. Despite this limitation, the proposed framework is designed in a way that is not inherently tied to a specific dataset. The use of multispectral features and physically interpretable vegetation indices provides a basis for potential transferability. However, further validation on diverse datasets acquired from different geographical regions, crop types, and environmental conditions is necessary to confirm its generalization capability.

While threshold-based and clustering methods can provide an initial approximation of stress regions, they rely on manually defined rules and fixed thresholds that may not generalize well under varying illumination, soil background, and crop conditions. In contrast, the proposed deep learning framework is capable of learning complex spatial and spectral patterns directly from the data, enabling more consistent and robust segmentation results. In addition, the model integrates contextual information and spatial coherence, which are difficult to capture using rule-based approaches alone.

Future work will focus on extending the dataset to include multiple geographical regions, crop types, and growth stages, as well as incorporating temporally diverse data to enable cross-scene and cross-region evaluations. In addition, manually annotated samples and hybrid labeling strategies will be integrated to further improve ground-truth quality and support a more rigorous assessment of model generalization and robustness under varying agricultural conditions.

## 7. Conclusions

This study presented WF-UNet++, a multi-head semantic segmentation framework with a physics-inspired FPDE-based output refinement module for harmonized crop stress mapping from heterogeneous UAV multispectral datasets. The proposed method allows consistent modeling of heterogeneous stress patterns across multi-source datasets by articulating rust disease and water stress inside a single five-class semantic space. With improvements of 6.8–7.7% in mIoU and 4.1–4.7% in Dice, experimental data show that the proposed framework outperforms cutting-edge CNN and transformer-based models. While the FPDE-based refinement module greatly increases spatial coherence, especially in fragmented and minority classes like high stress and rust disease, the multi-head architecture improves border localization and structural representation. With mIoU increasing from 0.7971 to 0.8623 in the final configuration, the ablation studies indicate that the combined integration of FPDE refinement, curriculum learning, class balancing, and auxiliary boundary-aware supervision improves the overall segmentation performance. From a methodological standpoint, incorporating a fixed-parameter, physics-inspired fractional-order diffusion refinement mechanism into the output probability space offers a rational means of enforcing spatial consistency beyond conventional post-processing approaches. The proposed architecture improves the representation of local structures and spatial continuity within the evaluated dataset setting when combined with edge-aware supervision and SDT-guided learning. Despite these benefits, there are a few drawbacks to be aware of. The tests may limit generalization across different regions and seasons because they were carried out on a small number of UAV orthomosaic datasets with comparable crop types and weather variables. Furthermore, compared to baseline models, the FPDE-based refinement adds more computational overhead, which could have an impact on real-time deployment in situations with limited resources. Future research will concentrate on enhancing computational efficiency, expanding the proposed framework to a variety of crop varieties and stress scenarios, and assessing it under cross-domain settings. Temporal modeling and semi-supervised learning could improve scalability even further and lessen reliance on labeled data. Furthermore, combining segmentation outputs with downstream activities like yield estimates and field-level decision systems continues to be a crucial path for real-world implementation.

## Figures and Tables

**Table 1:** Comparative summary of image-based segmentation studies for crop stress and disease analysis in maize.

Study	Crop & Scale	Target Phenomenon	Abiotic Stress	Biotic Disease	Task Type	Data Type	Classes/Granularity	Multi-Source Semantic Unification	Severity/Grading	Learning & Architecture	IoU/mIoU Reported
[19]	Maize, field	Weed–crop separation	✓ (weeds)	✗	Semantic segmentation	RGB	Crop + multi-weed	✗	✗	CNN-based segmentation	IoU ≈ 0.86 (class-wise)
[20]	Maize, leaf	GLS, NLB, NLS	✗	✓	Two-stage segmentation	RGB	Leaf/lesion	✗	✓	UNet + DeepLabv3+	mwIoU = 73.79%
[21]	Maize, UAV	Weed segmentation	✓	✗	Semantic segmentation	RGB	Crop/weed	✗	✗	CNN + transfer learning	IoU ≈ 0.82–0.88
[22]	Maize	Weed detection	✓	✗	Segmentation + detection	RGB	Crop/weed	✗	✗	Hybrid DL + OBIA	IoU not reported (R^2^/RMSE)
[23]	Maize, UAV	Rust severity	✗	✓	Monitoring (non-pixel)	Multi-view RGB	Severity indicators	✗	✓	Attention-based DL	IoU not reported (OA/F1)
[24]	Maize	Crop row structure	✗	✗	Classification	RGB	Row geometry	✗	✗	Anchor-based CNN	IoU not reported (F1 = 92.6%)
[25]	Multi-crop, leaf	Disease & damage	✗	✓	Segmentation + classification	RGB	Damage levels	✗	✓	CNN-based	IoU ≈ 0.78–0.83
[26]	Maize, UAV	Weed mapping	✓	✗	Semantic segmentation	RGB	Crop vs. weed	✗	✗	Semi-supervised CNN	mIoU ≈ 85.5%
[27]	Multi-crop (incl. maize)	Weed segmentation	✓	✗	Semantic segmentation	RGB	Crop/weed	✗	✗	Federated deep learning	mIoU ≈ 79.1%
[28]	Maize rows	Navigation guidance	✗	✗	Segmentation + geometry	RGB	Row/background	✗	✗	Lightweight CNN	IoU not reported (Accuracy used)
[29]	Maize, leaf-level	Leaf spot disease	✗	✓	Semantic segmentation	RGB	Disease mask	✗	✗	Attention + fusion UNet	mIoU = 92.62%
[30]	Multi-crop (incl. maize)	Leaf diseases	✗	✓	Semantic segmentation	Multi-modal	Disease regions	✗	✗	Fusion + graph CNN	IoU = 79.4%
[31]	Maize	Weed detection	✓	✗	Semantic segmentation	RGB	Crop mask	✗	✗	Knowledge-distilled CNN	mIoU > 95%
[32]	Maize, UAV	Straw residue cover	✓ (soil cover)	✗	Semantic segmentation	UAV RGB	Straw cover classes	✗	✓	Improved DeepLabv3+	mIoU ≈ 86–90%
[33]	Multi-crop UAV	Crop identification	✗	✗	Semantic segmentation	Multispectral	Crop types	✗	✗	Lightweight CNN	mIoU = 84.61%
[34]	Agricultural environment images	Agricultural scene understanding	✗	✗	Semantic segmentation	RGB (high-res field)	19 object classes (e.g., roads, obstacles, vegetation, etc.)	✗	✗	Multi-Scale Feature Alignment Network (MSFA-Net)	mIoU ≈ 84.46% (mPA ≈ 96.10%)
[35]	Maize canopy	Canopy segmentation	✓	✗	Semantic segmentation	RGB	Canopy/background	✗	✗	MCAC-UNet (Attention + ASPP)	mIoU = 87.51%
[36]	Maize leaf	Disease segmentation	✗	✓	Semantic segmentation	RGB	Lesion/background	✗	✓	YOLACT++ + Attention	mIoU = 84.91%
[37]	Maize field (UAV)	Straw/soil segmentation	✓	✗	Semantic segmentation	UAV RGB	Residue classes	✗	✓	Improved DeepLabv3+	mIoU = 93.97%
[38]	Maize field (UAV)	Crop segmentation	✗	✗	Semantic segmentation	UAV RGB	Crop/background	✗	✗	SegFormer/DeepLabv3+	mIoU = 78.81%
Proposed Study (This Work)	Maize, UAV multispectral orthomosaics	Plant health mapping	✓ (graded water stress)	✓ (rust)	Unified semantic segmentation	Multispectral UAV	5 classes (soil, healthy, low, high, rust)	✓	✓	UNet++ + multi-head + FPDE + curriculum	mIoU/FG-IoU = 0.8623/0.8439

**Table 2:** Comparative Evaluation of State-of-the-Art Segmentation Models and the Proposed Method.

Model	Best Epoch	Best Val IoU	Best Val IoU (FG)	Best Val Dice	Time (min)	Total Parameters
UNet	51	0.7945	0.7707	0.8836	16.0129	7,856,485
DeepLabV3+	42	0.7857	0.763	0.878	10.5311	7,856,485
WLUSNet [51]	33	0.7441	0.715	0.8506	7.7703	500,293
SwinUNet	52	0.7277	0.6897	0.8375	30.9117	12,437,317
SegFormer	52	0.7182	0.6789	0.8306	54.7037	4,407,749
HRNet-OCR	53	0.7014	0.6585	0.8166	25.4844	4,565,226
PSPNet	31	0.6665	0.6169	0.7875	11.4851	1,396,677
Mask2Former	25	0.3397	0.3149	0.4698	19.9151	7,961,413
Dual-PSPNet [19]	33	0.3343	0.292	0.4785	10.099	38,965,288
Proposed Method (WF-UNet++)	60	0.8623	0.8439	0.9250	186.7927	6,542,078

**Table 3:** Class-wise Pixel-Level F1 Scores of Compared Segmentation Models.

Model	F1 c0	F1 c1	F1 c2	F1 c3	F1 c4
UNet	0.9416	0.8711	0.8701	0.9237	0.8116
DeepLabV3+	0.9341	0.8684	0.8668	0.9212	0.7994
WLUSNet [51]	0.9250	0.8043	0.8288	0.9073	0.7875
SwinUNet	0.9359	0.7363	0.8009	0.9120	0.8024
SegFormer	0.9335	0.7216	0.7927	0.9092	0.7960
HRNet-OCR	0.9321	0.6676	0.7696	0.9123	0.8016
PSPNet	0.9275	0.5888	0.7242	0.9071	0.7898
Mask2Former	0.6101	0.0000	0.5574	0.7458	0.4358
Dual-PSPNet [19]	0.6697	0.1936	0.4458	0.6957	0.3876
Proposed Method (WF-UNet++)	0.9670	0.9340	0.9210	0.9430	0.8610

**Table 4:** Class-wise IoU results of the proposed method on the validation set (five classes).

Classes	IoU
Soil	0.9359
Healthy vegetation	0.8924
Low stress	0.8755
High stress	0.8522
Rust disease	0.7554
mIoU (Mean All Classes)	0.8623

**Table 5:** Overall segmentation performance metrics of the proposed method on the validation set.

Performance Metric	Value
FG-IoU	0.8439
Mean Dice	0.9250

**Table 6:** Component-Level Composition of the Proposed Model Across Ablation Stages.

Variation	Wavelet	Edge Head	SDT Head	FPDE	FPDE Curriculum	PatchBalance	BalancedMix
A	✗	✗	✗	✗	✗	✗	✗
B	✓	✗	✗	✗	✗	✗	✗
C	✓	✓	✗	✗	✗	✗	✗
D	✓	✓	✓	✗	✗	✗	✗
E	✓	✓	✓	✓	✗ (opens immediately)	✗	✗
F	✓	✓	✓	✓	✓ (with start/ramp)	✗	✗
G	✓	✓	✓	✓	✓	✓ (only “mix”)	✗
H	✓	✓	✓	✓	✓	✓ (only “mix”)	✓ (only “mix”)

**Table 7:** Quantitative ablation results of the proposed method on the validation set, including performance, training time, and model complexity.

Variation	Best Epoch	mIoU	FG-IoU	Mean Dice	Training Time (min)	Total Parameters
A	52	0.7971	0.7707	0.8853	91.06	6,518,265
B	65	0.8188	0.7962	0.8990	107.04	6,527,841
C	52	0.8077	0.7835	0.8920	94.40	6,534,954
D	52	0.8070	0.7828	0.8916	110.71	6,542,067
E	65	0.8153	0.7917	0.8968	110.39	6,542,078
F	52	0.8132	0.7894	0.8955	125.99	6,542,078
G	66	0.8421	0.8214	0.9131	170.53	6,542,078
H (Proposed Method) (WF-UNet++)	60	0.8623	0.8439	0.9250	186.7927	6,542,078

**Table 8:** Class-wise F1 Performance Across Ablation Variants (A–H).

Variation	F1 c0	F1 c1	F1 c2	F1 c3	F1 c4
A	0.948953	0.867904	0.868201	0.920653	0.820794
B	0.952470	0.891456	0.887193	0.927265	0.836451
C	0.949801	0.880102	0.878784	0.923998	0.827346
D	0.949463	0.876889	0.877959	0.924344	0.829487
E	0.952800	0.886102	0.882538	0.926275	0.836436
F	0.952158	0.884160	0.881286	0.926531	0.833333
G	0.960869	0.912677	0.902138	0.935173	0.854713
H (Proposed Method) (WF-UNet++)	0.9670	0.9340	0.9210	0.9430	0.8610

**Table 9:** Spatial summary of detected rust-affected zones derived from prediction results.

Zone ID	Class	Centroid (Lat, Lon)	Area (m^2^)
RR117	Rust	(39.342725, 33.949779)	255.9
RR132	Rust	(39.342692, 33.949214)	147.7
RR102	Rust	(39.342702, 33.949411)	60.0

**Table 10:** Spatial summary of detected water stress zones (low and high) derived from prediction results.

Zone ID	Class	Centroid (Lat, Lon)	Area (m^2^)
WH001	Water-High	(39.395465, 33.926412)	1064.2
WL001	Water-Low	(39.395403, 33.926469)	78.3
WL002	Water-Low	(39.395397, 33.926316)	70.3
WH002	Water-High	(39.395377, 33.926829)	61.9

**Figure A1:**
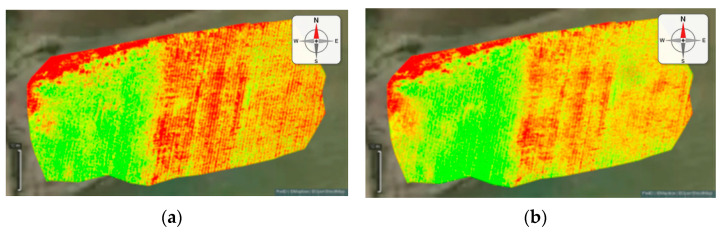

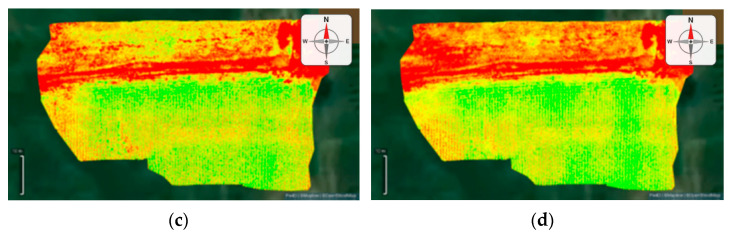
NDVI and NDRE maps used during the ground-truth generation process. The left column shows NDVI maps, while the right column presents NDRE maps. (**a**,**b**) correspond to the water-stress dataset, and (**c**,**d**) correspond to the rust-disease dataset.

## Data Availability

The raw data supporting the conclusions of this article will be made available by the authors on request. Code Sharing: The source code of the proposed WF-UNet++ framework will be publicly released on Kaggle and on the corresponding author’s GitHub account upon acceptance of the manuscript. Dataset availability: The multispectral orthomosaic datasets introduced in this study (“Water Stress 2025” and “Common Rust 2025”) will be made publicly available on Kaggle and on the corresponding author’s GitHub repository upon acceptance of the manuscript.

## Appendix A

Figure A1 provides supplementary spectral-index visualizations used during the rule-based ground-truth generation process. The NDVI maps support the interpretation of vegetation vigor and canopy degradation, whereas the NDRE maps provide additional sensitivity to chlorophyll-related stress responses. These maps are not presented as primary experimental results; instead, they serve as supporting evidence for the spectral-index-driven label construction procedure described in Section 3.3.1.

## References

[1] Houetohossou S.C.A., Houndji V.R., Hounmenou C.G., Sikirou R., Kakaï R.L.G. (2023). Deep Learning Methods for Biotic and Abiotic Stresses Detection and Classification in Fruits and Vegetables: State of the Art and Perspectives. Artif. Intell. Agric..

[2] Paul N., Sunil G., Horvath D., Sun X. (2025). Deep Learning for Plant Stress Detection: A Comprehensive Review of Technologies, Challenges, and Future Directions. Comput. Electron. Agric..

[3] Krizhevsky A., Sutskever I., Hinton G.E. (2012). Imagenet Classification with Deep Convolutional Neural Networks. Adv. Neural Inf. Process. Syst..

[4] LeCun Y., Bengio Y., Hinton G. (2015). Deep Learning. Nature.

[5] Long J., Shelhamer E., Darrell T. (2015). Fully Convolutional Networks for Semantic Segmentation. Proceedings of the IEEE Conference on Computer Vision and Pattern Recognition, Boston, MA, USA, 7–12 June 2015.

[6] Ronneberger O., Fischer P., Brox T. (2015). U-Net: Convolutional Networks for Biomedical Image Segmentation. Proceedings of the International Conference on Medical Image Computing and Computer-Assisted Intervention, Munich, Germany, 5–9 October 2015.

[7] Suiçmez Ç. (2025). Defect Segmentation of Magnetic Tiles with the Novel Ardise-U-Net. Trans. Electromagn. Spectr..

[8] Chen L.-C., Papandreou G., Kokkinos I., Murphy K., Yuille A.L. (2017). Deeplab: Semantic Image Segmentation with Deep Convolutional Nets, Atrous Convolution, and Fully Connected Crfs. IEEE Trans. Pattern Anal. Mach. Intell..

[9] Zhao H., Shi J., Qi X., Wang X., Jia J. (2017). Pyramid Scene Parsing Network. Proceedings of the IEEE Conference on Computer Vision and Pattern Recognition, Honolulu, HI, USA, 21–26 July 2017.

[10] Dyrmann M., Karstoft H., Midtiby H.S. (2016). Plant Species Classification Using Deep Convolutional Neural Network. Biosyst. Eng..

[11] Mortensen A.K., Dyrmann M., Karstoft H., Jørgensen R.N., Gislum R. Semantic Segmentation of Mixed Crops Using Deep Convolutional Neural Network. Proceedings of the CIGR-AgEng Conference.

[12] Sa I., Chen Z., Popovic M., Khanna R., Liebisch F., Nieto J., Siegwart R. (2017). Weednet: Dense Semantic Weed Classification Using Multispectral Images and Mav for Smart Farming. IEEE Robot. Autom. Lett..

[13] Milioto A., Lottes P., Stachniss C. Real-Time Semantic Segmentation of Crop and Weed for Precision Agriculture Robots Leveraging Background Knowledge in Cnns. Proceedings of the 2018 IEEE international conference on robotics and automation (ICRA).

[14] Chebrolu N., Lottes P., Schaefer A., Winterhalter W., Burgard W., Stachniss C. (2017). Agricultural Robot Dataset for Plant Classification, Localization and Mapping on Sugar Beet Fields. Int. J. Robot. Res..

[15] Pan S.J., Yang Q. (2010). A Survey on Transfer Learning. IEEE Trans. Knowl. Data Eng..

[16] Tuia D., Volpi M., Copa L., Kanevski M., Munñoz-Mari J. (2011). A Survey of Active Learning Algorithms for Supervised Remote Sensing Image Classification. IEEE J. Sel. Top. Signal Process..

[17] Wang M., Deng W. (2018). Deep Visual Domain Adaptation: A Survey. Neurocomputing.

[18] Das A., Pathan F., Jim J.R., Kabir M., Mridha M. (2025). Deep Learning-Based Classification, Detection, and Segmentation of Tomato Leaf Diseases: A State-of-the-Art Review. Artif. Intell. Agric..

[19] Picon A., San-Emeterio M.G., Bereciartua-Perez A., Klukas C., Eggers T., Navarra-Mestre R. (2022). Deep Learning-Based Segmentation of Multiple Species of Weeds and Corn Crop Using Synthetic and Real Image Datasets. Comput. Electron. Agric..

[20] Divyanth L., Ahmad A., Saraswat D. (2022). A Two-Stage Deep-Learning Based Segmentation Model for Crop Disease Quantification Based on Corn Field Imagery. Smart Agric. Technol..

[21] Gao J., Liao W., Nuyttens D., Lootens P., Xue W., Alexandersson E., Pieters J. (2023). Cross-Domain Transfer Learning for Weed Segmentation and Mapping in Precision Farming Using Ground and Uav Images. Expert Syst. Appl..

[22] Chen P., Xia T., Yang G. (2024). A New Strategy for Weed Detection in Maize Fields. Eur. J. Agron..

[23] Lv Z., Xu B., Zhong L., Chen G., Huang Z., Sun R., Huang W., Zhao F., Meng R. (2024). Improved Monitoring of Southern Corn Rust Using Uav-Based Multi-View Imagery and an Attention-Based Deep Learning Method. Comput. Electron. Agric..

[24] Quan L., Guo Z., Huang L., Xue Y., Sun D., Chen T., Geng T., Shi J., Hou P., He J. (2024). Efficient Extraction of Corn Rows in Diverse Scenarios: A Grid-Based Selection Method for Intelligent Classification. Comput. Electron. Agric..

[25] Polly R., Devi E.A. (2024). Semantic Segmentation for Plant Leaf Disease Classification and Damage Detection: A Deep Learning Approach. Smart Agric. Technol..

[26] Guo Z., Xue Y., Wang C., Geng Y., Lu R., Li H., Sun D., Lou Z., Chen T., Shi J. (2024). Efficient Weed Segmentation in Maize Fields: A Semi-Supervised Approach for Precision Weed Management with Reduced Annotation Overhead. Comput. Electron. Agric..

[27] Sivakumar S., Payyappilly A.J., Rajan A., Arun R.A., Rampriya R. (2025). A Computer Vision-Based Crop and Weed Segmentation Using Federated Ensemble Learning in Diverse Multi-Crop Fields. Eng. Appl. Artif. Intell..

[28] Luo Y., Gao G., Jiang P., Sun C., Xu M., Zhou Z., Hu W., Tan Y. (2025). A Navigation Line Extraction Method Based on Semantic Segmentation and Centerline Fitting. Smart Agric. Technol..

[29] Guo F., Yao C., Yang R., Ma M., Wu X., Xu Z., Lu M., Zhang J., Gong G. (2024). A High-Available Segmentation Algorithm for Corn Leaves and Leaf Spot Disease Based on Feature Fusion. Crop Prot..

[30] Hu Y., Wang G., Liu E., Zhu J., He M. (2025). Amf: A Multi-Modal Framework for Crop Leaf Diseases Segmentation. Comput. Electron. Agric..

[31] Liu T., Jin X., Han K., He F., Wang J., Chen X., Kong X., Yu J. (2024). Semantic Segmentation for Weed Detection in Corn. Pest Manag. Sci..

[32] Wang Y., Gao X., Sun Y., Liu Y., Wang L., Liu M. (2024). Semantic Segmentation-Based Conservation Tillage Corn Straw Return Cover Type Recognition. Comput. Electron. Agric..

[33] Mu Y., Wang H., Hu J., Sun Y., Zhu C., Zhu H., Ji Y., Gong H. (2025). Pcsnet: A Lightweight Semantic Segmentation Model for Low-Altitude Remote Sensing Mixed Crop Segmentation for Rapid Acquisition of Planting Information. Comput. Electron. Agric..

[34] Yao Z.-X., Wang H., Meng Z.-J., Yang L.-L., Zhang T.-H. (2026). Multi-Scale Feature Alignment Network for 19-Class Semantic Segmentation in Agricultural Environments. Artif. Intell. Agric..

[35] Gong H., Xiao L., Wang X. (2024). Segmentation and Coverage Measurement of Maize Canopy Images for Variable-Rate Fertilization Using the Mcac-Unet Model. Agronomy.

[36] Huang M., Xu G., Li J., Huang J. (2021). A Method for Segmenting Disease Lesions of Maize Leaves in Real Time Using Attention Yolact++. Agriculture.

[37] Liu Y., Zhang J., Wang Y., Luo Y., Sui P., Ren Y., Liu X., Wang J. (2025). Gcf-Deeplabv3+: An Improved Segmentation Network for Maize Straw Plot Classification. Agronomy.

[38] Martins J.A.C., Higuti A.Y.H., Pellegrin A.O., Juliano R.S., de Araújo A.M., Pellegrin L.A., Liesenberg V., Ramos A.P.M., Gonçalves W.N., Sant’ana D.A. (2024). Assessment of Uav-Based Deep Learning for Corn Crop Analysis in Midwest Brazil. Agriculture.

[39] Suicmez C. (2026). Developing New Artificial Intelligence-Based Models for the Detection of Diseases of Agricultural Products with Unmanned Aerial Vehicles. Ph.D. Thesis.

[40] Gao Q., Almekkawy M. (2021). Asu-Net++: A Nested U-Net with Adaptive Feature Extractions for Liver Tumor Segmentation. Comput. Biol. Med..

[41] Li J., Liu K., Hu Y., Zhang H., Heidari A.A., Chen H., Zhang W., Algarni A.D., Elmannai H. (2023). Eres-Unet++: Liver Ct Image Segmentation Based on High-Efficiency Channel Attention and Res-Unet++. Comput. Biol. Med..

[42] Mo Y., Wu Y., Yang X., Liu F., Liao Y. (2022). Review the State-of-the-Art Technologies of Semantic Segmentation Based on Deep Learning. Neurocomputing.

[43] Marmanis D., Schindler K., Wegner J.D., Galliani S., Datcu M., Stilla U. (2018). Classification with an Edge: Improving Semantic Image Segmentation with Boundary Detection. ISPRS J. Photogramm. Remote Sens..

[44] Chai D., Newsam S., Huang J. (2020). Aerial Image Semantic Segmentation Using Dcnn Predicted Distance Maps. ISPRS J. Photogramm. Remote Sens..

[45] Janev M., Pilipović S., Atanacković T., Obradović R., Ralević N. (2011). Fully Fractional Anisotropic Diffusion for Image Denoising. Math. Comput. Model..

[46] Kassimi S., Moussa H., Sabiki H. (2024). Enhancing Image Denoising: A Novel Non-Local Anisotropic Diffusion Framework Based on Caputo Derivatives and Gaussian Convolution for the Perona–Malik Model. Signal Process..

[47] Zhang W., Li J., Yang Y. (2015). Spatial Fractional Telegraph Equation for Image Structure Preserving Denoising. Signal Process..

[48] Huang Z., Wu G., Wang L. (2023). Webly-Supervised Semantic Segmentation Via Curriculum Learning. Comput. Vis. Image Underst..

[49] Hwang D., Kim H., Baek D., Kim H., Kye I., Choe J. (2025). Curriculum Learning with Class-Label Composition for Weakly Supervised Semantic Segmentation. Pattern Recognit. Lett..

[50] Kervadec H., Bouchtiba J., Desrosiers C., Granger E., Dolz J., Ben Ayed I. (2021). Boundary Loss for Highly Unbalanced Segmentation. Med. Image Anal..

[51] Zhu Q., Wang K., Liang D., Tang J. (2025). Wlusnet: A Lightweight Wheat Lodging Segmentation Network Based on Uav Image. Comput. Electron. Agric..

